# The Proteomic Landscape of Centromeric Chromatin Reveals an Essential Role for the Ctf19^CCAN^ Complex in Meiotic Kinetochore Assembly

**DOI:** 10.1016/j.cub.2020.10.025

**Published:** 2021-01-25

**Authors:** Weronika E. Borek, Nadine Vincenten, Eris Duro, Vasso Makrantoni, Christos Spanos, Krishna K. Sarangapani, Flavia de Lima Alves, David A. Kelly, Charles L. Asbury, Juri Rappsilber, Adele L. Marston

**Affiliations:** 1 The Wellcome Centre for Cell Biology, Institute of Cell Biology, School of Biological Sciences, Michael Swann Building, Max Born Crescent, Edinburgh EH9 3BF, UK; 2 Department of Physiology & Biophysics, University of Washington, Seattle, WA 98195-7290, USA; 3 Institute of Biotechnology, Technische Universität Berlin, Gustav-Meyer-Allee 25, 13355 Berlin, Germany

**Keywords:** meiosis, kinetochore, centromere, proteomics, Ctf19, CCAN, prophase I, metaphase I, budding yeast

## Abstract

Kinetochores direct chromosome segregation in mitosis and meiosis. Faithful gamete formation through meiosis requires that kinetochores take on new functions that impact homolog pairing, recombination, and the orientation of kinetochore attachment to microtubules in meiosis I. Using an unbiased proteomics pipeline, we determined the composition of centromeric chromatin and kinetochores at distinct cell-cycle stages, revealing extensive reorganization of kinetochores during meiosis. The data uncover a network of meiotic chromosome axis and recombination proteins that bind to centromeres in the absence of the microtubule-binding outer kinetochore sub-complexes during meiotic prophase. We show that the Ctf19c^CCAN^ inner kinetochore complex is essential for kinetochore organization in meiosis. Our functional analyses identify a Ctf19c^CCAN^-dependent kinetochore assembly pathway that is dispensable for mitotic growth but becomes critical upon meiotic entry. Therefore, changes in kinetochore composition and a distinct assembly pathway specialize meiotic kinetochores for successful gametogenesis.

## Highlights

• The composition of meiotic centromeres and kinetochores is revealed
• Kinetochores undergo extensive changes between meiotic prophase I and metaphase I
• The Ctf19^CCAN^ orchestrates meiotic kinetochore specialization
• A Ctf19^CCAN^-directed kinetochore assembly pathway is uniquely critical in meiosis

## Graphical Abstract

**Figure:**
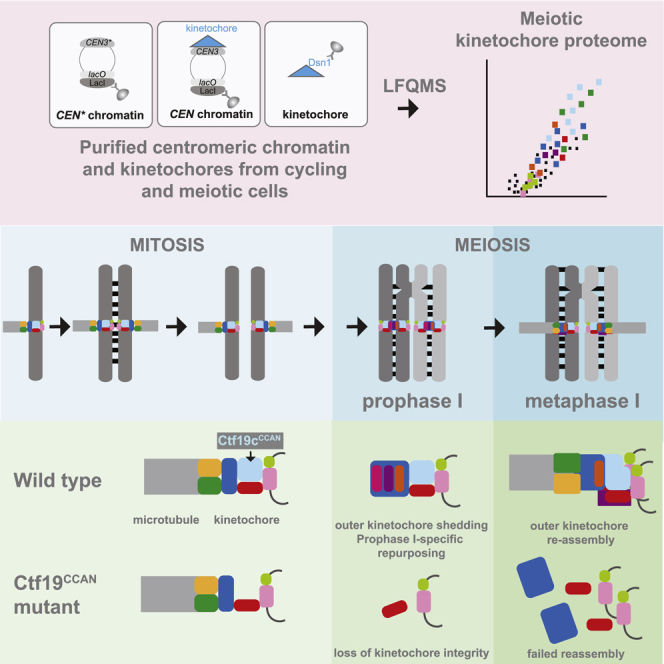

## Teaser

Kinetochores need to be specialized to direct the distinctive pattern of chromosome segregation in meiosis. Borek et al. reveal the protein composition of kinetochores in meiosis and identify a critical assembly pathway that promotes meiotic kinetochore specialization.

## Introduction

The kinetochore is a multi-molecular machine that links centromeric nucleosomes to microtubules for chromosome segregation.^1^ In budding yeast, sequence-specific binding of the Cbf3 complex (Cbf3c) enables formation of a single Cse4^CENP-A^-containing nucleosome,^2^ which directly contacts Mif2^CENP-C^ and components of the inner kinetochore 13-subunit Ctf19 complex (Ctf19c, known as CCAN in humans).^3^, ^4^, ^5^, ^6^, ^7^, ^8^, ^9^ Mif2^CENP-C^ and Ctf19c^CCAN^ form independent links to the 4-subunit Mtw1 complex (Mtw1c^MIS12c^, also known as MIND), forming the core of the kinetochore.^5^^,^^10^, ^11^, ^12^, ^13^ The outer Spc105 and Ndc80 complexes (Spc105c^KNL1c^ and Ndc80c^NDC80c^, respectively) assemble onto Mtw1c^MIS12c^ to provide the microtubule binding interface, which is stabilized by the Dam1 complex (Dam1c) in *S. cerevisiae* or the structurally distinct Ska complex in humans.^14^ A separate link from Ctf19c^CCAN^ to Ndc80c^NDC80c^, dispensable for viability in *S. cerevisiae*, is provided by Cnn1^CENP-T^.^15^, ^16^, ^17^

Kinetochores promote pericentromeric cohesin enrichment through cohesin loading onto centromeres,^18^, ^19^, ^20^, ^21^ shape pericentromere structure,^22^ and monitor proper attachment of chromosomes to microtubules.^23^ During meiosis, the specialized cell division that generates gametes, kinetochores adopt additional roles. These include non-homologous centromere coupling, repression of meiotic recombination, and co-segregation of sister chromatids in meiosis I.^24^ Uniquely during meiotic prophase, the outer kinetochore (Ndc80c^NDC80c^ and Dam1c) is shed, which may facilitate kinetochore specialization and recruitment of factors important for sister kinetochore monoorientation and cohesin protection.^25^, ^26^, ^27^ Meiotic kinetochore defects have been implicated in age-related oocyte deterioration in humans, causing infertility, birth defects, and miscarriages.^28^

Many functions of the kinetochore require Ctf19c^CCAN^, yet only the Ame1^CENP-U^-Okp1^CENP-Q^ heterodimer is essential for growth. Though viable, cells lacking other Ctf19c^CCAN^ subunits show chromosome segregation defects, which may, in part, be attributed to their role in loading centromeric cohesin.^18^^,^^20^^,^^21^ The N-terminal extension of Ctf19^CENP-P^ recruits the cohesin loading complex,^29^ while the Ctf19^CENP-P^ C-terminal domain is a receptor for the Ipl1^AURORA B^ kinase.^9^^,^^30^ The Ctf19c^CCAN^ is further implicated in various meiosis-specific processes, including the non-homologous coupling of centromeres in early meiotic prophase and suppression of crossover recombination near centromeres.^31^^,^^32^

Here, we use quantitative proteomics to determine budding yeast kinetochore and centromere composition in meiotic prophase I, metaphase I, and mitotically cycling cells, uncovering adaptations for meiosis. We demonstrate a specific requirement for Ctf19c^CCAN^ in kinetochore function in early meiosis, revealing an assembly pathway that is uniquely essential for kinetochore organization in gametogenesis.

## Results

### Chromatin, Centromere, and Kinetochore Proteomes

**Figure 1:**
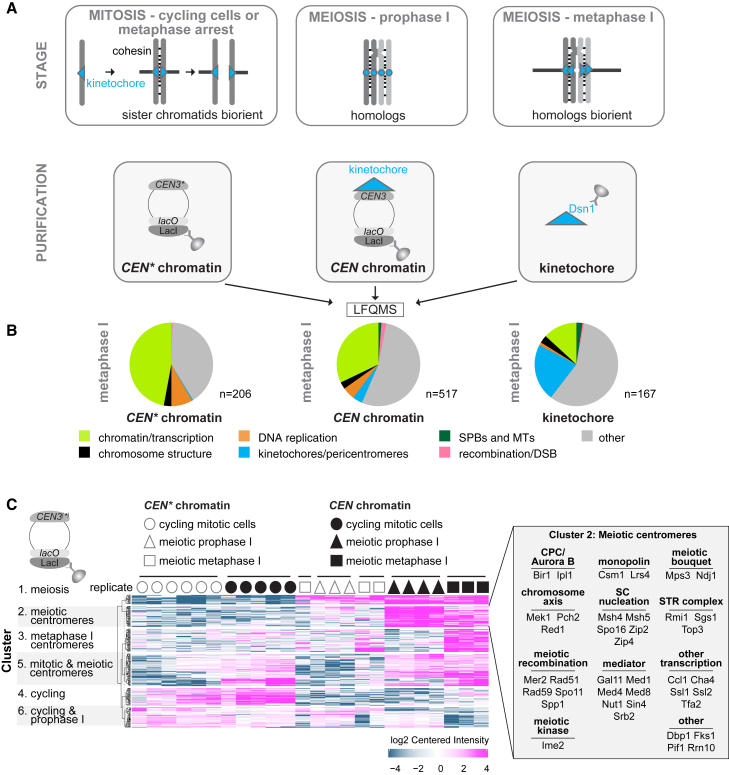
Quantitative Label-Free Mass Spectrometry (LFQMS) Reveals the Complexity of the Centromere and Kinetochore-Associated Proteomes (A) Schematic representation of determined proteomes. *CEN* chromatin, *CEN^∗^* chromatin, and kinetochores were isolated from cycling, prophase I-arrested, and metaphase I-arrested cells and subjected to LFQMS. (B) *CEN^∗^* chromatin, *CEN* chromatin, and kinetochores show respective increases and decreases in the fraction of enriched proteins that are associated with chromatin or kinetochores. Following immunoprecipitation of LacI-3FLAG (*CEN* chromatin and *CEN^∗^* chromatin) and Dsn1-6His-3FLAG (kinetochores), proteins were quantified using LFQMS, and those enriched over respective negative controls with a cut-off of Log_2_(fold change) > 4 and p < 0.01 were categorized in the indicated groups. (C) Stage-specific functional groups of proteins associating with *CEN* chromatin and *CEN^∗^* chromatin. k-means clustering with a cut-off of Log_2_(fold change) > 2 and p < 0.05 was used. Cluster 2 proteins are listed in the inset. See also Table S1.

To reveal the changes in centromeric chromatin composition that underlie its specialized functions during meiosis, we analyzed the proteome of minichromosomes isolated from budding yeast cells at different cell-cycle stages. We immunoprecipitated LacI-FLAG bound to *lacO* arrays on a circular minichromosome carrying the budding yeast centromere 3 (*CEN3*) sequence (Figure 1A; *CEN* chromatin).^33^ To identify chromatin-associated proteins that require a functional centromere, in parallel, we analyzed the proteome of a minichromosome that is identical except for two mutations within *CEN3* that abolish formation of the centromeric nucleosome and therefore prevent kinetochore assembly (Figure 1A; *CEN^∗^* chromatin).^33^ Using label-free quantitative mass spectrometry (LFQMS), we compared the composition of *CEN* and *CEN^∗^* chromatin in three conditions: mitotically cycling cells, cells arrested in meiotic prophase I (by deletion of *NDT80*, encoding a global meiotic transcription factor), and cells arrested in meiotic metaphase I (by depletion of the APC/C activator, Cdc20). We also generated an orthogonal LFQMS dataset by direct immunoprecipitation of FLAG-tagged core kinetochore protein, Dsn1^DSN1^,^34^ representing cycling mitotic, meiotic prophase I, meiotic metaphase I cells, and additionally, cells arrested in mitotic metaphase by treatment with the microtubule-depolymerizing drug benomyl (Figure 1A). Meiotic prophase I and metaphase I arrests were confirmed by measuring the completion of DNA replication and spindle morphology, respectively (Figures S1A and S1B).

In all three datasets (*CEN^∗^* chromatin, *CEN* chromatin, and kinetochore), chromatin-associated proteins were highly enriched over a no-tag control (Figure 1B). In metaphase I, *CEN* chromatin contained the largest number of specifically enriched proteins (517), while kinetochore proteins formed the largest and smallest fraction of the kinetochore and *CEN^∗^* proteomes, respectively (Figure 1B). *CEN* and *CEN^∗^* chromatin from mitotically cycling cells were more similar to each other than to the meiotic samples and the presence of a centromere affected meiotic chromatin composition more than stage (Figure 1C; Table S1). Groups of proteins enriched on meiotic chromatin (cluster 1), meiotic centromeres (cluster 2), or metaphase I centromeres (cluster 3) corresponded to the expected functional categories. For example, cluster 2, showing meiotic and centromere-dependent enrichment (Figure 1C), included proteins involved in centromere coupling, initiation of synapsis, kinetochore monoorientation, and the chromosome passenger complex (CPC). The kinetochore proteome (Figures S1C and S1D; Table S2) similarly clustered into groups enriched in meiosis (KTcluster 1), or specifically at either metaphase I (KTcluster 2) or prophase I (KTcluster 5). Arrest in mitosis resulted in a kinetochore proteome that was remarkably similar to that of cycling cells, except for expected increases in spindle checkpoint proteins (Mad1^MAD1^, Mad2^MAD2^, Bub1^BUB1^, and Bub3^BUB3^), cohesin, and Cdc5^Plk1^, and a decrease in the Mcm2–7 replicative helicase (Figure S1E), suggesting little variation in kinetochore composition throughout the mitotic cell cycle. Therefore, *CEN* and kinetochore proteomics detect cell-cycle-dependent changes in chromatin, centromere, and kinetochore composition.

### Chromatin, Centromere, and Kinetochore Composition Changes during Meiosis

Comparison of prophase I and metaphase I *CEN^∗^* chromatin with that of cycling cells revealed enrichment of the meiosis-specific cohesin subunit, Rec8, and depletion of mitosis-specific Scc1 (Figures S2A and S2B). Meiotic axis (Hop1, Red1) and synaptonemal complex-nucleating ZMM (Zip1^SYCP1^-Zip2^SHOC1^-Zip3^RNF212^-Zip4^TEX11^, Msh4^MSH4^-Msh5^MSH5^, Mer3^HFM1^) proteins were also enriched in prophase I, together with the STR dissolvase (Sgs1^BLM^, Top3^TOPIIIα^, Rmi1^RMI1/RMI2^), consistent with their roles in meiotic recombination and synapsis.^35^^,^^36^

**Figure 2:**
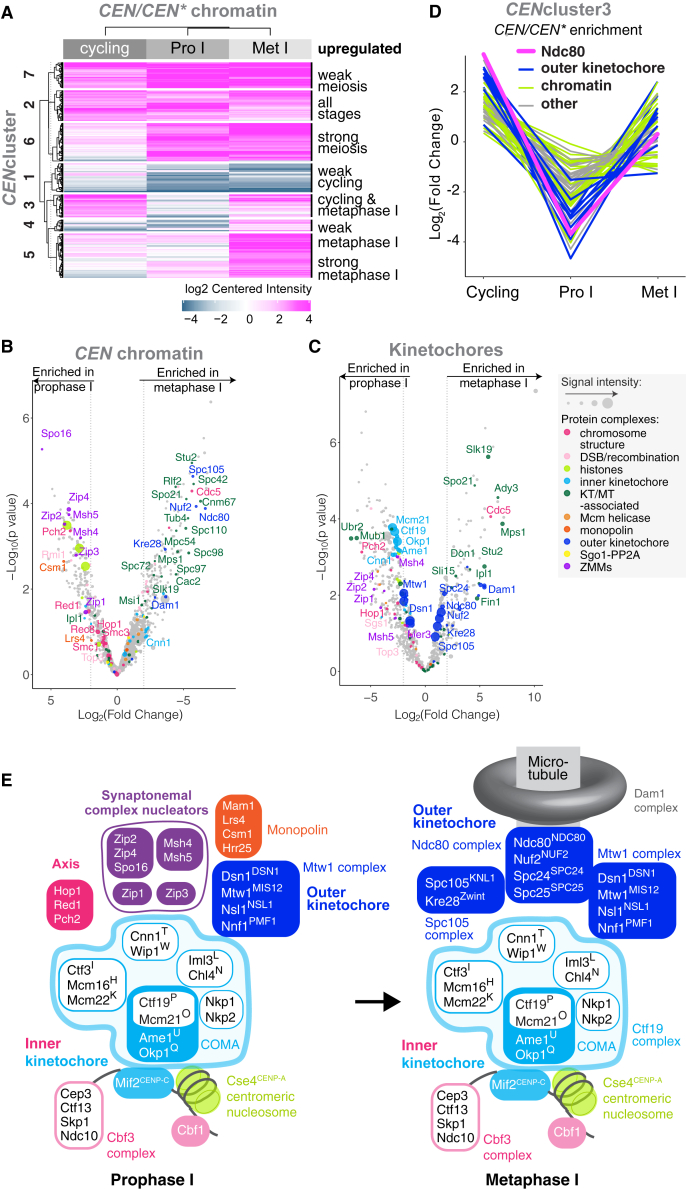
Changes in the Centromeric and Kinetochore Proteomes between Meiotic Prophase I and Metaphase I (A) *CEN* chromatin exhibits distinct composition signatures at different stages. The *CEN* chromatin/*CEN^∗^* chromatin enrichment values for each of cycling, prophase I-arrested, and metaphase I-arrested conditions were clustered (k-means) to identify groups of proteins with similar behavior. A cut-off of Log_2_(fold change) > 2 and p < 0.05 was used. (B and C) Composition of *CEN* chromatin (B) or kinetochore particles (C) isolated from prophase I and metaphase I is strikingly different. Volcano plot presenting the LFQMS-identified proteins co-purifying with *CEN* plasmids (B) or Dsn1-6His-3FLAG (C) immunopurified from cells arrested in prophase I (*ndt80*Δ, B; inducible*-NDT80*, C) and metaphase I (*pCLB2-CDC20*). Log_2_(fold change) between conditions is shown with corresponding p values (STAR Methods). Dashed line indicates |Log_2_(fold change)| = 2. (D) Several proteins exhibit Ndc80^NDC80^-like depletion from centromeres specifically during meiotic prophase. Mean-centered Log_2_(fold change) from *CEN*cluster3 is plotted to show abundance of individual proteins in the indicated stages. (E) Schematic illustrating changes in the kinetochore association of some key complexes between meiotic prophase I and metaphase I observable by proteomics. See also Figures S1 and S2 and Tables S2 and S3.

Changes in the protein composition of centromeres and kinetochores during meiosis were revealed by clustering only those proteins that specifically associate with functional centromeres (*CEN* and not *CEN^∗^*; Figure 2A; Table S3). *CEN*cluster 6 proteins show increased centromere association during meiosis and include the ZMM proteins. *CEN*cluster 5 proteins are specifically enriched on meiotic metaphase I centromeres and include Cdc5^Plk1^ kinase, which is recruited to kinetochores at prophase I exit to establish monoorientation.^37^ *CEN*cluster 3 proteins associate with centromeres of cycling and meiotic metaphase I cells, but are depleted at prophase I. Consistently, this cluster included outer kinetochore complexes Ndc80c^NDC80c^ and Dam1c, which are shed at prophase I due to specific degradation of Ndc80^NDC80^ protein^25^^,^^38^^,^^39^ (Table S3). Direct comparison of the prophase I and metaphase I datasets revealed extensive changes in the composition of kinetochores upon prophase exit (Figures 2B and 2C). Zip1^SYCP1^ together with SZZ (Spo16^SPO16^, Zip2^SHOC1^, Zip4^TEX11^) and Msh4^MSH4^-Msh5^MSH5^ complexes are lost from *CEN* chromatin and kinetochores as cells transition from prophase I to metaphase I. Conversely, outer kinetochore proteins (Ndc80c^NDC80c^ and Dam1c), spindle pole body components, and microtubule-associated proteins are recruited in metaphase I. Spc105^KNL1^ and its binding partner Kre28^Zwint^ were also specifically depleted from prophase I *CEN* chromatin and kinetochores, returning in metaphase I (Figures 2B and 2C). In contrast, both Mtw1c^MIS12c^ and Ctf19c^CCAN^ associated with kinetochores at all analyzed stages (Figures S2C and S2D). Although *CEN* and kinetochore purifications were largely comparable, chromatin assembly factor I (CAF-I, Cac2-Mri1-Rlf2) associated specifically with metaphase I *CEN* chromatin (Figure 2B), while Ubr2-Mub1, known to regulate Dsn1^DSN1^ stability in mitotic cells,^40^ was found only on prophase I kinetochore preparations (Figure 2C).

Plotting the relative abundance of proteins in *CEN*cluster3 revealed that multiple proteins were depleted from centromeric chromatin during prophase I, similar to Spc105c^KNL1c^, Ndc80c^NDC80c^, and Dam1c (Figure 2D; Table S3). These include the Ndc80c^NDC80c^-associated PP1 phosphatase regulator, Fin1, the microtubule regulator Stu2^XMAP215^^33^^,^^41^, and several chromatin regulators. The schematic in Figure 2E summarizes the kinetochore association of selected complexes detected by proteomics to highlight changes in kinetochore composition between meiotic prophase I and metaphase I.

### Centromeric Cohesion Establishment Is Not the Only Essential Function of Ctf19c^CCAN^ in Meiosis

The changes in centromeric chromatin and kinetochores during meiosis suggest the existence of specialized assembly mechanisms. Ctf19c^CCAN^ may play a critical role in this process because Ctf19c^CCAN^ proteins that are dispensable for vegetative growth are essential for chromosome segregation during meiosis, and Ctf19^CENP-P^ is implicated in meiotic kinetochore assembly.^18^^,^^42^ Indeed, we confirmed that Ctf19c^CCAN^ mutant cells show a moderate loss of viability during vegetative growth, while the completion of meiosis and spore survival is drastically reduced (Figures S3A–S3D). Consistently, mitotic nuclei divide evenly in Ctf19c^CCAN^ mutant cells, while during meiosis, nuclear division is highly aberrant (Figures S3E–S3G).

**Figure 3:**
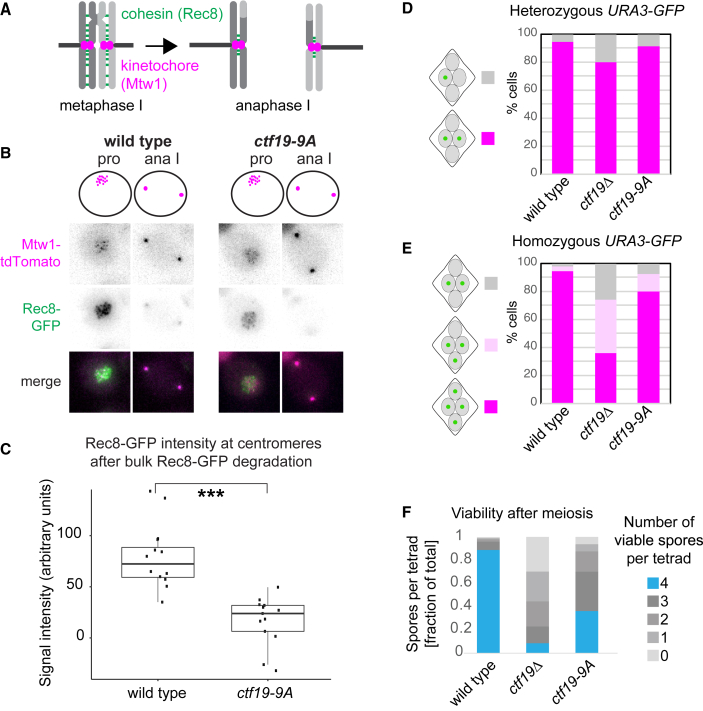
Pericentromeric Cohesion Is Absent in *ctf19-9A* Anaphase I Cells (A–C) Pericentromeric cohesin is reduced in *ctf19-9A* anaphase I cells. Wild-type and *ctf19-9A* cells expressing Rec8-GFP and Mtw1-tdTomato were imaged throughout meiosis. (A) Schematic showing Rec8^REC8^ loss from chromosome arms, but not pericentromeres in anaphase I. (B) Representative images are shown. (C) Quantification of Rec8-GFP signal in the vicinity of Mtw1-tdTomato foci immediately following bulk Rec8-GFP degradation. Whiskers represent 1.5 IQR (interquartile range), the middle line is median, and the box encompasses two middle quartiles of the data. ^∗∗∗^p < 10^−5^; Mann-Whitney test. n > 11 cells. (D and E) *ctf19-9A* cells show less severe meiosis II chromosome segregation defects than *ctf19Δ* cells. The percentage of tetra-nucleate cells with the indicated patterns of GFP dot segregation was determined in wild-type and *ctf19-9A* cells with either one copy (heterozygous, D) or both copies (homozygous, E) of chromosome V marked with GFP at *URA3* locus. n = 2 biological replicates, 100 tetrads each; mean values are shown. (F) Spore viability of *ctf19-9A* cells is impaired, but less severe than *ctf19Δ* cells. The number of viable progeny was scored following tetrad dissection. n = 3 biological replicates, >70 tetrads each; mean values are shown. See also Figure S3.

The Ctf19c^CCAN^ directs cohesin loading at centromeres, establishing robust pericentromeric cohesion to ensure accurate sister chromatid segregation during meiosis II.^19^, ^20^, ^21^^,^^32^ Ctf19c^CCAN^ mutants also mis-segregate chromosomes during meiosis I,^18^^,^^42^ suggesting Ctf19c^CCAN^ plays additional meiotic roles. The *ctf19-9A* mutation, which abolishes centromeric cohesin loading, but preserves kinetochore function,^29^ fails to retain Rec8-containing cohesin at pericentromeres following anaphase I (Figures 3A–3C). Interestingly, segregation of GFP-labeled sister chromatids during meiosis II and spore viability were impaired in *ctf19-9A* cells to a lesser extent than in the *ctf19Δ* mutant (Figures 3D–3F). Therefore, although Ctf19^CENP-P^-directed cohesin loading is crucial, other essential functions of Ctf19c^CCAN^ exist in meiosis.

### Central Role of Ctf19c^CCAN^ in Meiotic Kinetochore Organization

**Figure 4:**
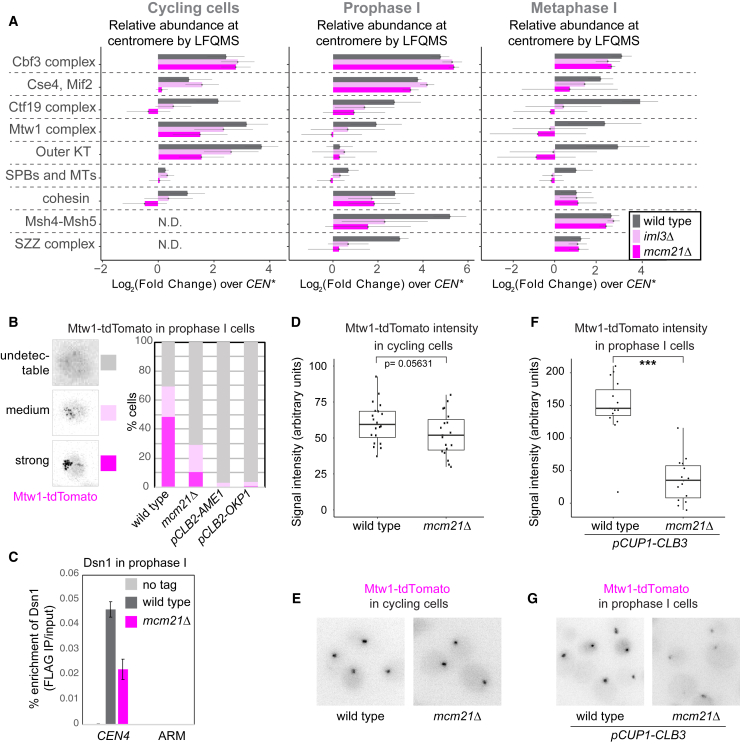
Proteomics Identifies a Critical Role of Ctf19c^CCAN^ in Meiotic Kinetochore Assembly (A) Global *CEN/CEN^∗^* proteomics reveals that kinetochore composition is altered in *mcm21Δ* and *iml3Δ* meiotic prophase I and metaphase I cells. The sum of LFQMS abundance of protein complexes on *CEN* chromatin in wild-type, *iml3*Δ, and *mcm21*Δ cells is shown as enrichment over *CEN^∗^* chromatin isolated from wild-type cells. The abundance of Iml3 ^CENP-L^ and Mcm21^CENP-O^ proteins was not included in the total Ctf19c^CCAN^ count, as these proteins are missing in *iml3*Δ and *mcm21*Δ cells, respectively (STAR Methods). Error bars represent SD. KT, kinetochore; MT, microtubule; SPB, spindle pole body; SZZ, Spo16^SPO16^, Zip2^SHOC1^, Zip4^TEX11^. (B–G) A functional Ctf19c^CCAN^ is critical for Mtw1c^MIS12c^ association with centromeres in meiotic prophase I, but not cycling cells. (B) Wild-type, *mcm21Δ*, *pCLB2-AME1*, and *pCLB2-OKP1* cells were imaged immediately after release from prophase I arrest. Representative images and scoring of cells with Mtw1-tdTomato signal are shown. n > 58 cells. (C) Prophase I-arrested wild-type and *mcm21*Δ cells carrying *ndt80Δ* and *DSN1-6His-3FLAG*, together with untagged control, were subjected to anti-FLAG ChIP-qPCR. Error bars represent SE (n = 4 biological replicates). p < 0.05, paired t test. (D–G) Mtw1-tdTomato signal intensity in cycling (D and E) and prophase I-arrested (F and G) wild-type and *mcm21*Δ cells. In (F) and (G), cells were engineered to ectopically produce Clb3 to maintain kinetochore clustering and allow signal quantification. In (D) and (F), whiskers represent 1.5 IQR, the middle line is median, and the box encompasses the two middle quartiles of the data. ^∗∗∗^p < 10^−5^; Mann-Whitney test. n > 19 (D) or n = 15 cells (F). See also Figure S4.

To understand how Ctf19c^CCAN^ affects meiotic kinetochore composition, we exploited our *CEN* chromatin proteomics pipeline, focusing on two Ctf19c^CCAN^ subunits, Mcm21^CENP-O^ and Iml3^CENP-L^. Cycling, prophase I, and metaphase I *mcm21Δ* and *iml3Δ CEN* chromatin datasets showed significant deviations in composition from wild type, affecting multiple core and associated kinetochore complexes (Figures 4A, S4A, S4B, S1A, and S1B). In cycling *mcm21Δ* and *iml3Δ* cells, as expected,^5^ there was an overall reduction in Ctf19c^CCAN^, mostly due to loss of the non-essential subunits, rather than the essential subunits, Ame1^CENP-U^ and Okp1^CENP-Q^ (Figures S4A and S4B, Cycling cells). The abundance of the central Mtw1c^MIS12c^ and outer Ndc80c^NDC80^ was also modestly decreased on *CEN* chromatin from cycling *mcm21Δ* and *iml3Δ* cells (Figures 4A, S4A, and S4B, Cycling cells). Cse4^CENP-A^-Mif2^CENP-C^ appeared reduced on *CEN* chromatin from *mcm21*Δ cells (Figures 4A and S4A); however, this may be a consequence of their low kinetochore abundance, which precludes consistent detection by mass spectrometry. *CEN* chromatin of cycling *mcm21Δ* and *iml3Δ* mutants additionally bound less cohesin, consistent with a failure to load it at centromeres in these cells.^18^^,^^19^^,^^29^ At prophase I, the outer kinetochore components Ndc80c^NDC80c^, Dam1c, and Spc105c^KNL1c^ were depleted from wild-type, *mcm21Δ*, and *iml3Δ CEN* chromatin, as expected (Figures 4A, S4A, and S4B, Prophase I). Interestingly, enrichment of Msh4^MSH4^-Msh5^MSH5^ and SZZ complexes with *CEN* chromatin in prophase I required Mcm21^CENP-O^ and Iml3^CENP-L^. Furthermore, Mtw1c^MIS12c^ was lost from *mcm21Δ* and *iml3Δ* prophase I and metaphase I *CEN* chromatin, and Ndc80c^NDC80c^, Dam1c, and Spc105c^KNL1c^ did not reappear in metaphase I (Figures 4A, S4A, and S4B, Metaphase I). Consistently, microtubule-associated and spindle pole body proteins were recovered with wild-type, but not *iml3Δ* or *mcm21Δ*, metaphase I *CEN* chromatin, while the DNA-binding Cbf3c was not affected (Figure 4A). We conclude that the Ctf19c^CCAN^ plays a major role in ensuring the integrity of the kinetochore in meiotic prophase, its reassembly upon prophase I exit, and kinetochore reorganization in meiosis.

### Ctf19c^CCAN^ Retains Mtw1c^MIS12c^ at Prophase I Kinetochores

Our *CEN* proteomics shows that, surprisingly, Mtw1c^MIS12c^ is lost from prophase I kinetochores in *mcm21Δ* and *iml3Δ* cells. In mitotic cells, a version of Ame1^CENP-U^ that is unable to bind and localize Mtw1c^MIS12c^ to kinetochores does not support growth.^5^ To determine whether loss of Mtw1c^MIS12c^ could explain the severe meiotic phenotypes of *mcm21Δ* and *iml3Δ* cells, we visualized Mtw1-tdTomato during meiosis. At meiotic prophase I, kinetochores de-cluster and appear as up to 16 individual foci, each representing a pair of homologous centromeres (Figure 4B). Strong or medium Mtw1-tdTomato foci were detected in ∼70% of wild-type prophase I cells, but only ∼30% of *mcm21Δ* cells and less than 5% of Ame1^CENP-U^- or Okp1^CENP-Q^-depleted cells (Figure 4B). Similarly, chromatin immunoprecipitation followed by qPCR (ChIP-qPCR) showed that Dsn1^DSN1^ was significantly reduced at prophase I centromeres of *mcm21Δ* cells (Figure 4C).

Moreover, Mcm21^CENP-O^ is critical for Mtw1-tdTomato recruitment to kinetochores in meiosis, but not mitosis. Cycling *mcm21Δ* cells show only a modest decrease in Mtw1-tdTomato signal intensity as compared to wild type (Figures 4D and 4E). In contrast, prophase I-arrested *mcm21Δ* cells engineered to preserve kinetochore clustering (by overexpression of cyclin *CLB3*, which prevents outer kinetochore shedding^25^) showed Mtw1-tdTomato signal intensity that was barely above background (Figures 4F and 4G). Therefore, the kinetochore association of Mtw1c^MIS12c^ in meiosis requires not only Ctf19c^CCAN^ subunits Ame1^CENP-U^- and Okp1^CENP-Q^, as in mitosis, but additionally Mcm21^CENP-O^ and Iml3^CENP-L^, unlike in mitosis.

### Mtw1c^MIS12c^ Loss in Early Meiosis in Ctf19c^CCAN^ Mutants Precludes Chromosome Segregation in the Subsequent Division

**Figure 5:**
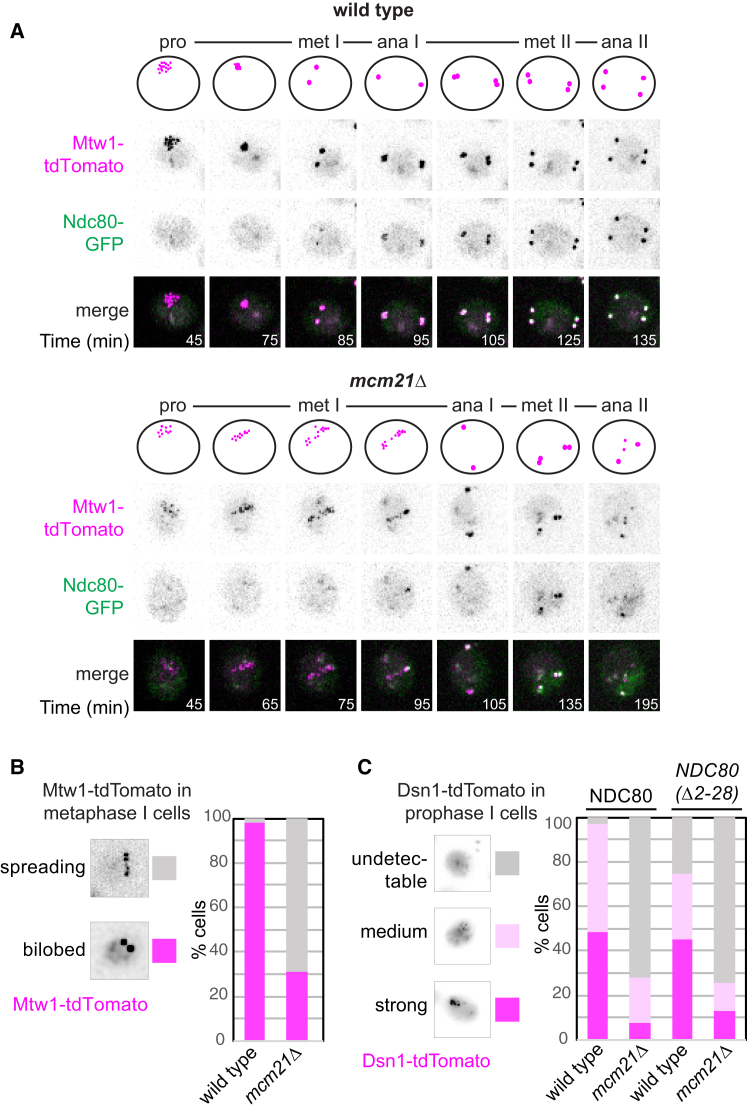
An Intact Ctf19c^CCAN^ Is Required for Functional Outer Kinetochore Assembly in Meiosis I (A and B) Abnormal kinetochore behavior in the absence of *MCM21.* (A) Representative images of wild-type and *mcm21*Δ cells carrying Mtw1-tdTomato and Ndc80-GFP after release from prophase I arrest and imaged throughout meiosis. Time after release from prophase I is indicated. (B) Scoring of Mtw1-tdTomato signal in (A). Cells showing kinetochore spreading in at least one time point during the time-lapse were included in the “spreading” category. n > 49 cells. (C) Non-degradable *ndc80(Δ2-28)* does not rescue kinetochore function upon the loss of *MCM21.* Dsn1-tdTomato signal was scored in prophase I wild-type and *mcm21*Δ cells expressing either Ndc80-GFP or Ndc80(Δ2-28)-GFP. n > 56 cells. See also Figure S5.

At prophase I exit in wild-type cells, Ndc80^NDC80^ is re-synthesized leading to outer kinetochore re-assembly and re-attachment to microtubules.^25^^,^^38^^,^^43^ In *mcm21Δ* cells, however, Ndc80-GFP re-accumulation at kinetochores was delayed, and foci were fainter (Figure 5A). Where detected in *mcm21Δ* cells, individual Mtw1-tdTomato kinetochore foci tended to “spread,” rather than form bilobed clusters typical of metaphase I (Figure 5B), suggesting that residual outer kinetochore re-assembly is insufficient to support microtubule attachment. Consistently, prevention of outer kinetochore shedding by using a non-degradable allele of *NDC80*^38^ did not restore Dsn1-tdTomato kinetochore localization in *mcm21Δ* cells (Figure 5C; see also Figures 4F and 4G). Therefore, kinetochore disintegration in *mcm21Δ* and *iml3Δ* meiotic cells is not a consequence of programmed outer kinetochore loss during prophase I.

We tested whether Mtw1c^MIS12c^ loss occurring in meiotic prophase I can be rescued if the subsequent division is mitosis, rather than meiosis. Meiotic prophase I-arrested cells were induced to re-enter the mitotic program by addition of nutrients (Figure S5A) and Mtw1-tdTomato was observed in the ensuing mitotic metaphase. Following nutrient addition, wild-type cells re-clustered kinetochores and budded, and the Mtw1-tdTomato focus split into two before segregating into the daughter cells, as expected.^44^ In contrast, in the majority of *mcm21Δ* cells, Mtw1-tdTomato foci were initially undetectable; subsequently, around the time of bud emergence, weak foci appeared, splitting into daughter cells with a substantial delay (Figure S5B). Kinetochore spreading at mitotic metaphase was more apparent in those *mcm21Δ* cells where Mtw1-tdTomato was initially undetectable (Figures S5C–S5E). Consistently, *mcm21Δ* cells show decreased viability after return to growth (Figure S5F). Therefore, prior to completion of prophase I, kinetochores undergo a precipitous and irreversible event that relies upon the presence of the Ctf19c^CCAN^ to ensure correct chromosome segregation.

### Ctf19c^CCAN^ Is Critical for Kinetochore-Microtubule Attachments in Meiosis I

**Figure 6:**
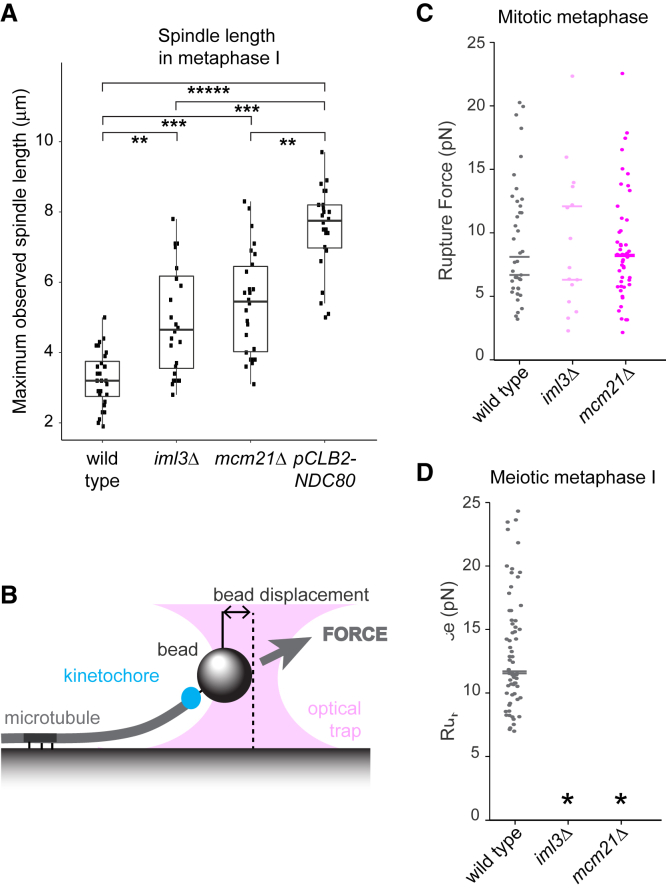
Essential Role for Ctf19c^CCAN^ in Establishment of Kinetochore Microtubule Attachments in Meiosis I (A) Metaphase I spindles are elongated in *iml3Δ* and *mcm21*Δ cells. Wild-type, *iml3*Δ, *mcm21*Δ, and *pCLB2-NDC80* cells carrying *pCLB2-CDC20* and expressing Mtw1-tdTomato and GFP-Tub1 were imaged undergoing meiosis and the maximum observed spindle length was measured. Whiskers represent 1.5 IQR, the middle line is median, and the box encompasses the two middle quartiles of the data. ^∗∗∗∗∗^p < 10^−15^, ^∗∗∗^p < 10^−7^, ^∗∗^p < 10^−4^; t test. n > 23. (B–D) Purified kinetochore particles (Dsn1-6His-3FLAG immunoprecipitation) from *iml3Δ* and *mcm21Δ* cells fail to attach to microtubules in a single-molecule assay. (B) Schematic of assay showing the optical trap pulling on a bead attached to a coverslip-immobilized microtubule. The bead-microtubule interaction is facilitated by purified kinetochores. (C and D) Kinetochore particles isolated from metaphase I-arrested cells lacking *IML3* and *MCM21* are not able to form kinetochore-microtubule attachments *in vitro*. Rupture force measurements of kinetochore particles isolated from mitotically arrested (by the addition of benomyl, C) or meiosis metaphase I-arrested (due to the presence of *pCLB2-CDC20*, D) wild-type, *iml3*Δ, and *mcm21*Δ cells are shown. Total particles analyzed: n = 65 (wild type, meiosis), n = 41 (wild type, mitosis), n = 15 (*iml3Δ*, mitosis), and n = 49 (*mcm21Δ*, mitosis) from 2 biological replicates; bars represent medians for each replicate. Asterisks indicate conditions for which no initial kinetochore-microtubule attachment was formed and thus rupture force could not be measured. See also Figure S6 and Table S7.

The spreading of kinetochore foci along a linear axis (Figures 5A and 5B) and the extended duration of meiosis I in *mcm21Δ* cells (Figure S6A) suggest defective attachment of kinetochores to microtubules, leading to engagement of the spindle checkpoint. Consistently, both degradation of the anaphase inhibitor, Pds1^SECURIN^, and cleavage of cohesin are delayed in *mcm21Δ* and *iml3Δ* cells (Figures S6B–S6D). Furthermore, metaphase I spindles were longer in *mcm21Δ* and *iml3Δ* cells than in wild-type cells, but shorter than in Ndc80^NDC80^-depleted cells, which lack kinetochore-microtubule attachments (Figure 6A). This indicates that kinetochore-microtubule attachments are impaired, but not completely absent, in meiotic cells lacking Ctf19c^CCAN^. To address this directly, we purified kinetochores from both mitotic and meiotic metaphase I wild-type, *mcm21Δ*, and *iml3Δ* cells (using Dsn1-6His-3FLAG immunoprecipitation) and assayed their ability to resist laser trap forces after binding to microtubules growing from a coverslip-anchored seed (Figure 6B).^34^^,^^45^ Wild-type kinetochore particles from mitotic metaphase cells bound microtubules with a mean rupture force of ∼9 pN, and this was unchanged for kinetochore particles purified from *mcm21Δ* or *iml3Δ* mitotically cycling cells (Figure 6C). Wild-type meiotic metaphase I kinetochore particles showed an increased rupture force (mean ∼12 pN), as reported previously,^45^ whereas both *mcm21Δ* and *iml3Δ* kinetochore particles completely failed to bind to microtubules (Figure 6D). Therefore, purified metaphase I kinetochores from *mcm21Δ* and *iml3Δ* cells fail to make load-resisting attachments to microtubules, suggesting that the changes in kinetochore composition are sufficient to explain the gross chromosome segregation defects *in vivo*. The more severe kinetochore-binding defect of *mcm21Δ* and *iml3Δ* kinetochores *in vitro* (Figure 6D) than *in vivo* (Figure 5A) is likely a consequence of the purification procedure, which can expose kinetochore vulnerabilities not observed *in vivo*.^15^

### Ctf19c^CCAN^ Maintains Kinetochore Integrity Early in Gametogenesis

Why are meiotic kinetochores so critically dependent on Ctf19c^CCAN^? Cnn1^CENP-T^, which links the inner and outer kinetochore, is lost from *mcm21Δ* and *iml3Δ* mitotic and meiotic kinetochores (Figure S7A),^5^ but *cnn1Δ* cells show no apparent meiotic chromosome segregation defects (Figure S7B). Similarly, loss of kinetochore integrity in *mcm21Δ* meiotic cells is not caused by the need to accommodate monopolin, since Mtw1-tdTomato association is not rescued by deletion of the monopolin component *MAM1* (Figure S7C).

**Figure 7:**
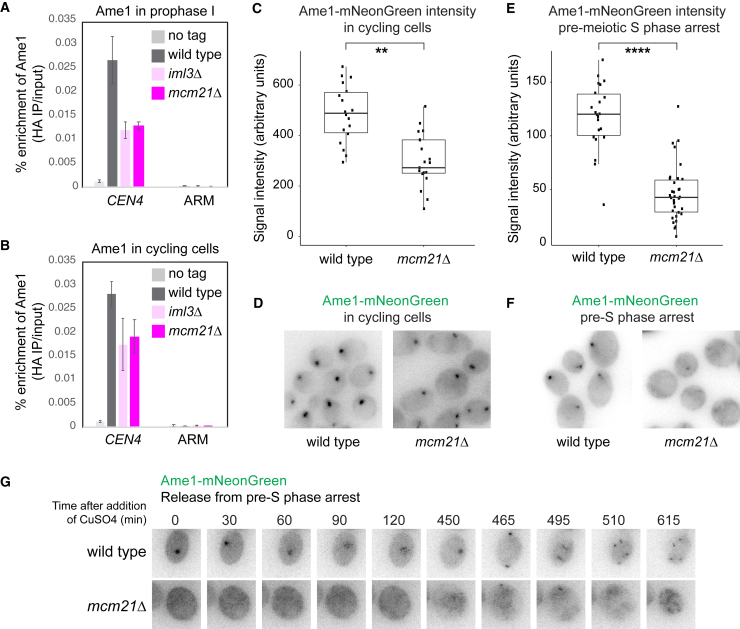
Ctf19c^CCAN^ Subunits that Are Dispensable for Mitosis Become Essential for Inner Kinetochore Retention upon Entry into Gametogenesis (A and B) Loss of essential inner kinetochore component Ame1^CENP-U^ in cycling and prophase I cells lacking *IML3* and *MCM21*. Prophase I-arrested (A) or cycling (B) wild-type, *iml3Δ*, and *mcm21*Δ cells carrying *AME1-6HA*, together with untagged control, were subjected to anti-HA ChIP-qPCR. Error bars represent SE (n = 3 or 4 biological replicates, cycling and prophase I-arrested cells, respectively). (C–F) Ame1-mNeonGreen imaging in cycling (C and D) and pre-S phase-arrested (E and F) wild-type and *mcm21*Δ cells. Signal quantification (C and E) and representative images (D and F) are shown. In (C) and (E), whiskers represent 1.5 IQR, the middle line is median, and the box encompasses the two middle quartiles of the data. ^∗∗∗∗^p < 10^−10^, ^∗∗^p < 10^−4^; t test. n > 13 (C) or n > 17 cells (E). (G) Wild-type and *mcm21*Δ cells expressing Ame1-mNeonGreen were allowed to synchronously enter pre-meiotic S-phase through induction of *IME1* and *IME4*, followed by live-cell imaging. See also Figure S7.

Instead, the common effects of *MCM21* deletion and Ame1^CENP-U^ or Okp1^CENP-Q^ depletion on the association of Mtw1c^MIS12c^ with the kinetochore (Figure 4B) suggested that Mcm21^CENP-O^ might become more important for localization of Ame1^CENP-U^-Okp1^CENP-Q^ at kinetochores in meiosis. This was supported by the observation that *CEN* chromatin purified from metaphase I-arrested cells showed a substantial loss of the Ame1^CENP-U^-Okp1^CENP-Q^ heterodimer in *mcm21Δ* and, to a lesser extent, *iml3Δ* cells as compared to wild type (Figure S4A, Metaphase I). ChIP-qPCR found that Ame1^CENP-U^ levels were reduced approximately 2-fold at endogenous centromeres of *mcm21Δ* and *iml3Δ* prophase I cells and, unexpectedly, were also reduced in cycling cells, albeit to a slightly lesser extent (Figures 7A and 7B). Because cross-linking during ChIP may stabilize dynamic Ame1^CENP-U^ at centromeres in *mcm21Δ* and *iml3Δ* prophase I cells, we also performed live-cell imaging. This revealed that while in cycling cells only a fraction of total Ame1-mNeonGreen was lost from *mcm21Δ* kinetochores, as compared to wild type (Figures 7C and 7D), it was nearly undetectable in *mcm21*Δ cells arrested before pre-meiotic S phase (by blocking expression of the meiotic master regulators, Ime1 and Ime4^46^^,^^47^) (Figures 7E and 7F), or after release from this arrest (Figure 7G). Together, these observations suggest a dynamic turnover of Ame1^CENP-U^ at *mcm21Δ* meiotic kinetochores.

Kinetochore catastrophe is not simply a consequence of starvation because Ame1-mNeonGreen persisted upon abrupt transfer of actively cycling cells to sporulation medium (which is not compatible with immediate entry into meiosis) (Figure S7D). Therefore, upon initiation of the meiotic program, Ctf19c^CCAN^ subunits that are dispensable for mitotic growth become crucial for localizing the components of the Ctf19c^CCAN^ that are essential for viability.

Ame1^CENP-U^ is not the only essential kinetochore protein lost from meiotic kinetochores in *mcm21Δ* cells. Remarkably, though below the detection limit of proteomics (Figure 4A), centromeric levels of Mif2^CENP-C^ (Figure S7E), Cse4^CENP-A^ (Figure S7F), and the DNA-binding component of the Cbf3c, Ndc10 (Figures S7G and S7H), were also decreased in meiotic *mcm21Δ* cells. Overall, we find that the entire meiotic kinetochore, including its DNA-binding components, is heavily impacted by loss of the non-essential Ctf19c^CCAN^ subunits, while only modest effects on mitotic kinetochores are observed.

## Discussion

Meiotic kinetochores perform a myriad of functions that are essential for healthy gamete formation, from homolog pairing and spatial regulation of meiotic recombination in prophase I, to the establishment and monitoring of oriented attachments to microtubules in metaphase I. Our global analysis of centromeric chromatin and kinetochores provides a framework for understanding how their meiosis-specific functions are executed and regulated. This approach to document chromatin composition in meiosis could be adapted to study other genetic loci of interest, such as recombination hotspots and replication origins.

### Specialization of Kinetochores for Meiosis-Specific Functions

Our data highlight the re-purposing of kinetochores in meiotic prophase when microtubule-binding elements of the outer kinetochore (Ndc80c^NDC80c^ and Dam1c) are absent.^25^^,^^27^^,^^38^ Proteomics suggested that, though detectable by live-cell imaging,^39^ Spc105c^KNL1c^ is also diminished at prophase I kinetochores. This implies that loss of Ndc80c^NDC80c^ weakens Spc105c^KNL1c^ interaction with the inner kinetochore, reducing its recovery by immunoprecipitation. We find that in the absence of the Ndc80c^NDC80c^ and Dam1c, the inner kinetochore Ctf19c^CCAN^ and the central Mtw1c^MIS12c^ serve as a platform for assembly of prophase I-specific regulators. These include meiotic axis proteins, the STR dissolvase, and the ZMM pro-crossover and synaptonemal complex (SC) nucleation factors (Figure S4). Potentially, the centromeric localization of these factors plays a role in preventing crossover formation within pericentromeres, though the pro-crossover properties of ZMMs would need to be silenced. Notably, the crossover-promoting factors MutLγ (Mlh1-Mlh3), which associate with ZMMs at presumed crossover sites on chromosome arms,^48^^,^^49^ were not found at meiotic centromeres, suggesting that regulating their recruitment may restrict crossover formation. Our data also reveal that the monopolin complex, which binds directly to the Dsn1^DSN1^ subunit of Mtw1c^MIS12c^ and directs kinetochore monoorientation during meiosis I,^50^^,^^51^ associates with centromeres already in meiotic prophase I, while Cdc5^Plk1^, another key regulator of monoorientation,^52^^,^^53^ associates with kinetochores later in meiosis I. Therefore, extensive re-organization during meiotic prophase establishes kinetochore functionality that persists into the meiotic divisions.

### Central Role of the Ctf19c^CCAN^ in Defining Meiotic Kinetochores

Through a global proteomics approach and single-cell imaging, we identified a central role for the Ctf19c^CCAN^ in reorganizing kinetochores for meiosis. Ctf19c^CCAN^ is critical both for preventing pericentromeric crossovers^31^ and for maintaining cohesive linkages between sister chromatids until meiosis II.^18^^,^^32^^,^^42^ Targeted Ctf19c^CCAN^-dependent cohesin loading at centromeres contributes to both pericentromeric crossover suppression^31^ and meiosis II chromosome segregation (Figures 3D–3F). However, we find that defective pericentromeric cohesin does not fully explain the profound meiotic chromosome segregation defects observed in Ctf19c^CCAN^-deficient meiosis. Indeed, multiple key regulators are lost from meiotic kinetochores lacking Ctf19c^CCAN^ components, such as SZZ and Msh4^MSH4^-Msh5^MSH5^ complexes. Future work will be required to determine whether these proteins are recruited by the Ctf19c^CCAN^ directly and whether they play a role in any of its meiotic functions.

We also found that Ctf19c^CCAN^ acts in a kinetochore assembly pathway that is uniquely essential in meiosis. We show that in the absence of Ctf19c^CCAN^ subunits that are dispensable for viability, the Mtw1c^MIS12c^, Ndc80c^NDC80c^, Spc105c^KNL1c^, and Dam1 complexes together with the entire Ctf19c^CCAN^ are all lost from meiotic centromeres, resulting in a failure of chromosomes to attach to microtubules, catastrophic segregation errors, and inviable gametes. We suggest that the near-complete absence of kinetochore proteins assembled on centromeric DNA in Ctf19c^CCAN^-deficient cells may also underlie a cohesin-independent function of Ctf19c^CCAN^ in preventing double-strand break formation near centromeres.^31^

### A Distinct Kinetochore Assembly Pathway that Is Critical for Meiosis

Why are meiotic kinetochores so critically dependent on the Ctf19c^CCAN^ while mitotic cells can survive in the complete absence of most of its subunits? Our findings suggest that non-essential Ctf19c^CCAN^ subunits contribute to kinetochore integrity in cycling cells too, albeit to much a lesser extent than in meiosis. This implies that the Ctf19c^CCAN^-directed kinetochore assembly pathway that is critical for meiosis also functions in cycling cells where it is non-essential, presumably due to the existence of redundant assembly mechanisms. Whether the predominant use of Ctf19c^CCAN^-directed kinetochore assembly pathway in meiosis is coupled to the functional specialization of kinetochores for the unique meiosis I chromosome segregation pattern remains to be determined. Alternatively, differences in cell-cycle wiring may underlie the greater importance of the Ctf19c^CCAN^-directed pathway in meiosis. Budding yeast kinetochores remain attached to microtubules throughout the mitotic cell cycle, except for a brief period during S phase.^54^ In contrast, meiotic kinetochores remain partially disassembled during a prolonged S phase and prophase I and are subsequently rebuilt later in meiosis. This is similar to the mammalian mitotic cell cycle, in which CCAN directs the sequential assembly of MIS12c and NDC80c as cells progress from interphase into mitosis.^55^ Such altered kinetochore turnover in meiosis may expose a vulnerability that leads to more stringent requirements for kinetochore assembly, potentially manifest as a critical dependence on Ctf19c^CCAN^.

Interestingly, Ctf19^CENP-P^ is a receptor for Ipl1^AURORA B^ at inner kinetochores in mitotic cells^9^^,^^30^ and Aurora B-dependent phosphorylation of Dsn1^DSN1^ is known to facilitate stable kinetochore assembly.^12^^,^^56^, ^57^, ^58^, ^59^ Furthermore, Ipl1^AURORA B^ plays several meiosis-specific functions including triggering outer kinetochore shedding and preventing premature spindle assembly in prophase I.^26^^,^^39^^,^^60^ We speculate that recruitment of Ipl1^AURORA B^ is the critical role of Ctf19c^CCAN^ in meiosis, though future work will be required to determine the molecular and structural details of kinetochore reorganization during meiosis. Our comprehensive analysis of meiotic kinetochore composition provides an extensive resource for the discovery of these mechanisms.

## STAR★Methods

### Key Resources Table

**Table:** 

REAGENT or RESOURCE	SOURCE	IDENTIFIER
**Antibodies**		

Mouse anti-Ha (HA11)	BioLegend	MMS-101R; RRID: AB_291262
Mouse anti-Ha (12CA5)	Roche	11583816001; RRID: AB_514505
Mouse anti-FLAG M2	Sigma	F1804; RRID: AB_262044
Rabbit anti-Pgk1	Lab stock	N/A
Rabbit anti-Myc (9E10)	Covance/Biolegend	626802; RRID: AB_2148451
Sheep anti-mouse HRP	GE Healthcare	NXA931; RRID: AB_772209
Donkey anti-rabbit HRP	GE Healthcare	NA934; RRID: AB_772206
Rat anti-tubulin	Bio-Rad	MCA77G; RRID: AB_325003
Donkey anti-rat FITC	Jackson ImmunoResearch	712-095-153; RRID: AB_2340652

**Chemicals, Peptides, and Recombinant Proteins**		

b-estradiol	Sigma	E2758
Benzonase	Merck	71206-3
Chelex 100	Bio-Rad	1422822
Proteinase K	Life Technologies	25530049
Dynabeads	ThermoFisher	10009D
Trypsin	Pierce	90057
NuPage LDS Sample buffer	ThermoFisher	NP0008
Chymostatin	Melford	C1104
Leupeptin (Hemisulphate)	Melford	L1001
E64	Melford	E1101
Pepstatin A	Melford	P2203
Antipain, dihydrochloride	Melford	A0105
Aprotinin	Melford	A2301
AEBSF hydrochloride 98%	ACROS Organics	32811010
N-Ethylmaleimidine 99+%	ACROS Organics	156100050
COmplete-EDTA-free tablets	Roche	11873580001
Microcystin-L	LKT Laboratories	M3406
Zymolyase	AMS Biotechnology	120491-1
Yeast nitrogen base	Formedium	CYN0410
CSM -Trp +20 Ade dropout medium	Formedium	DCS0269
Dimethyl Pimelimidate	Sigma	D8388
Rapigest	Waters	186001861
NuPAGE Novex 4-12% Bis-Tris gel	ThermoFisher	NP0321BOX

**Deposited Data**		

The LFQMS data have been deposited to the ProteomeXchange Consortium via the PRIDE partner repository.	This study	PPXD019754
The interactive visualizations of LFQMS data have been deposited to the University of Edinburgh datashare.	This study	https://doi.org/10.7488/ds/2916

**Experimental Models: Organisms/Strains**		

Yeast strains used in this study	N/A	See Table S4

**Oligonucleotides**		

Oligonucleotides used in this study	N/A	See Table S6

**Recombinant DNA**		

Plasmids used in this study	N/A	See Table S5

### Resource Availability

#### Lead Contact

Further information and request for resources and reagents should be directed to and will be filled by the Lead Contact, Adele Marston: adele.marston@ed.ac.uk

#### Materials Availability

Yeast strains and plasmids generated in this study are available without restriction through the lead contact.

#### Data and Code Availability

The mass spectrometry proteomics data have been deposited to the ProteomeXchange Consortium via the PRIDE partner repository ^61^ with the dataset identifier PRIDE: PXD019754. Interactive volcano plots for comparison of different conditions are available for download as .html files from https://doi.org/10.7488/ds/2916.

### Experimental Model and Subject Details

#### Yeast strains and plasmids

All yeast strains are SK1 derivatives and are listed in Table S4. Plasmids generated in this study are listed in Table S5. Gene deletions, promoter replacements and gene tags were introduced using standard PCR-based methods, with the exception of the *CSE4-mNeonGreen* and *ndc80*(*Δ2-28*)*-GFP*^38^ alleles that were generated by CRISPR (see below). *DSN1-6His-3FLAG*,^45^ *pCLB2-CDC20*,^52^ inducible-*NDT80 (pGAL1-NDT80*, *pGPD1-GAL4.ER*^62^), *ndt80Δ*,^31^ *CEN5*-GFP dots,^63^ *PDS1-tdTomato* and *HTB1-mCherry*,^64^ *pCUP1-IME1/pCUP1-IME4*^47^ were described previously.

#### CSE4-mNeonGreen

Cse4 was internally tagged with mNeonGreen by inserting the fluorescent tag flanked by two long linkers into the long N-terminal tail of Cse4, between L81 and E82. mNeonGreen flanked by linkers was amplified from AMp1604 (pFA6a-mNeonGreen-KlLEU2) using primers each with 100 bp homology to *CSE4* (AMo8738, AMo8660). Primers encoding sgRNA (AMo7441, AMo7442) allowing a Cas9 cut at *CSE4* G79 were cloned into AMp1278 (pWS082^65^) to produce AMp1295. The sgRNA encoding fragment was amplified from AMp1295 using primers AMo6663, AMo6664 (guide: AGCAGGTAATCTAGAAATCG). A fragment containing Cas9 and a *URA* marker was amplified from AMp1279 (pWS158^65^) with primers AMo6723, AMo6724. All three fragments were transformed into yeast and correct integrants confirmed by sequencing. Primer sequences are given in Table S6.

#### *NDC80*(*Δ2-28*)*-GFP* construction

A fragment of AMp1362 (3xV5-*NDC80Δ2-28*, *LEU2*, kind gift from Elçin Unal^38^) was amplified using primers AMo6819, AMo6853. A fragment containing Cas9 and a *URA* marker was amplified from AMp1279 (pWS158^65^) with primers AMo6723, AMo6724. Primers encoding sgRNA (AMo6847, AMo6846) allowing a Cas9 cut at *NDC80* M15 were cloned into AMp1278 (pWS082^65^) to produce AMp1467. The sgRNA encoding fragment was amplified from AMp1467 using primers AMo6663, AMo6664 (guide TCAACATGTGCTACATCACA). All three fragments were transformed into a strain carrying *NDC80-GFP* and correct integrants confirmed by sequencing. Primer sequences are given in Table S6.

#### Yeast carrying *CEN* and *CEN^∗^* minichromosomes

Plasmids AMp1103 (pSB963; *CEN3*, *8xlacO*, *TRP1*) and AMp1106 (pSB972; *CEN3^∗^*, *8xlacO, TRP1*)^33^ were amplified by PCR, digested with *EcoR*I to remove sequences required for propagation in *E. coli*,^66^ re-ligated and the ∼2kb minichromosomes were transformed into haploid SK1 strains carrying integrated Stu1-cut AMp747 (pSB737; LacI-3FLAG, *URA3*.^33^ Diploids of the appropriate genotype were generated by mating.

### Method Details

#### Yeast growth conditions

##### Meiotic Induction

To obtain meiotic cultures (apart from those used for *CEN/CEN*^∗^ IPs, described below), yeast strains were grown at 30°C for 16 h on YPG plates (1% yeast extract, 2% bactopeptone, 2.5% glycerol, and 2% agar) and then for 8 – 24 h on YPD4% plates (1% yeast extract, 2% bactopeptone, 4% glucose, and 2% agar). Cells were then cultured in YPDA (1% yeast extract, 2% bactopeptone, 2% glucose, 0.3 mM adenine) overnight, and then inoculated at an OD_600_ = 0.2 – 0.5 into BYTA (1% yeast extract, 2% bactotryptone, 1% potassium acetate, 50 mM potassium phthalate) and grown overnight to an OD_600_ ≥ 3. Cells were harvested, washed twice with an equal volume of water, resuspended into SPO medium (0.3% potassium acetate) to an OD_600_ ≥ 1.9 and incubated at 30°C with vigorous shaking. For metaphase I arrest, a meiotic shut-off allele of *CDC20* was used (*pCLB2- CDC20*)^52^ with harvesting of cells 8 h after resuspension in SPO medium. For prophase I arrest, *ndt80Δ*^67^ or inducible-*NDT80* was used and cells were fixed or harvested 5–6 h after resuspension in SPO medium. For synchronous meiosis, inducible-*NDT80* was used to allow prophase I block-release, as described by Carlile and Amon.^68^ For synchronization at meiotic entry, *pCUP1-IME1 pCUP1-IME4* allele was used, as described.^46^

##### Cycling cells and mitotically arrested cultures for kinetochore purifications

To harvest mitotically cycling cells, overnight cultures were diluted to OD_600_ = 0.1, in YPDA and harvested by centrifugation at approximately OD_600_ = 1.0. Cells were washed once with ice-cold dH_2_O, then washed twice with 50 mL ice-cold dH_2_O supplemented with 0.2 mM PMSF. dH_2_O supplemented with 0.2 mM PMSF was added to 15% volume of the pellet, mixed and drop frozen into liquid nitrogen before storage at −80°C.

To harvest mitotic cultures for Dsn1-6His-3FLAG immunoprecipitation, 300 μg/mL benomyl was added to 1.9 L of boiling YEP (1% yeast extract, 2% bactopeptone) and allowed to cool, before adding glucose to 2% and adenine to 0.3 mM. An overnight starter culture in YPDA was diluted into 4 × 500 mL of YPDA to OD_600_ = 0.3 in 4 L flasks and grown to OD_600_ = 1.6 – 1.8, before adding 500 mL of cooled benomyl-containing YPDA to each flask.

##### Growing strains for *CEN* and *CEN^∗^* immunoprecipitation

Cryo-stored diploids were grown on YPG for 16 h, then inoculated into 50 mL of liquid YPDA and shaken overnight at 250 rpm at 30°C. After ∼20 – 24 h, the 50 mL of YPDA culture (OD_600_ ≥ 10) was transferred to 200 mL of -TRPA medium (see below) in a 2 L flask and shaken at ≥200 rpm overnight. The following day ∼2 pm, 60 mL of -TRPA culture was added to each of four 2 L flasks containing 450 mL of -TRPA medium and shaken at ≥ 200 rpm overnight at 30°C. The following morning, cultures with OD600 ≥ 5 were spun for 5 – 8 min at 4 – 5 krpm in a Beckmann centrifuge rotor 91000, and washed twice with dH_2_O at room temperature (wash 1: 1 L, wash 2: 0.5 L). The pellet was resuspended in 150 mL of SPO medium (0.3% potassium acetate), and 50 mL of this cell suspension added to each of three 4 L flasks containing 450 mL of SPO medium. Cells were then grown for 5–6 h, spun, drop-frozen and stored at −80°C until needed.

-TRPA medium was adapted from Suhandynata et al.^69^ and was made by dissolving 28 g of yeast nitrogen base (Formedium) mixed with 16 g of -TRP dropout powder (Formedium, CSM -Trp +20 Ade) in 900 mL of water. Following autoclaving, 12.5 mL sterile-filtered solution of glucose (40%) was added to 0.5% final concentration, and 25 mL potassium acetate (0.8 g/mL) was added to 2% final concentration. The solution was topped-up with sterile water to 1 L.

##### Induction of *CLB3* expression in meiotic cells

25 μM copper sulfate was added after 3 h to meiotic cultures of wild-type and *mcm21*Δ cells harboring *pCUP1-CLB3* allele. Two h later, cells were released from the prophase I arrest.

#### Immunoprecipitation

##### Preparation of anti-FLAG conjugated Dynabeads

Protein G Dynabeads (500 μL; Invitrogen) were washed twice in 1 mL 0.1M Na-phosphate, pH 7.0, before incubating with 50 μL M2 anti-FLAG monoclonal antibody (SIGMA) and 50 μL of 0.1M Na-phosphate with gentle agitation for 30 min at room temperature. Beads were washed twice in 1 mL of 0.1 M Na-phosphate pH 7.0 with 0.01% Tween 20, then washed twice with 1 mL of 0.2 M triethanolamine, pH 8.2. Antibody-conjugated Dynabeads were resuspended in 1 mL of 20 mM DMP (Dimethyl Pimelimidate, D8388, Sigma) in 0.2 M triethanolamine, pH 8.2 (prepared immediately before use) and incubated with rotational mixing for 30 min at room temperature. Beads were concentrated, the supernatant removed and 1 mL of 50 mM Tris-HCl, pH 7.5 added before incubating for 15 min with rotational mixing. The supernatant was removed and beads were washed three times with 1 mL 1XPBST+0.1% Tween-20 before resuspending in 300 mL of 1xPBST.

##### Immunoprecipitation of *CEN/CEN^∗^* chromatin

Yeast cells were pulverised mechanically using a Retsch RM100 mortar-grinder.

Approximately 20 g of cryogrindate was used per experiment. Cryogrindates were resuspended in H_0.15_ buffer (25 mM HEPES (pH 8.0), 2 mM MgCl_2_, 0.1 mM EDTA (pH 8.0), 0.5 mM EGTA-KOH (pH 8.0), 15% glycerol, 0.1% NP-40, 150 mM KCl) supplemented with phosphatase inhibitors (2 mM β-glycerophosphate, 1 mM Na_4_P_2_O_7_, 5 mM NaF, 0.1 mM Na_3_VO_4_), protease inhibitors (2 mM final AEBSF, 0.2 μM microcystin and 10 μg/mL each of ‘CLAAPE’ protease inhibitors (chymostatin, leupeptin, antipain, pepstatin, E64)) and 1 mM N-Ethylmaleimide (NEM) at 1 g of grindate to 1.5 mL of complete buffer ratio. Debris was removed by centrifugation (1x5 min at 5 krpm, 1x15 min at 5 krpm) and lysates incubated at 4°C for 3 h with Protein G Dynabeads previously conjugated to mouse anti-Flag (M2, Sigma) with DMP (Dimethyl Pimelimidate, D8388, Sigma). 12.5 μL of bead suspension and 5.8 μL of antibody were used per 1 g of grindate. Beads were washed three times in H_0.15_ buffer before sequential elution at 37°C for 2x30 min in 1% Rapigest (Waters).

##### Immunoprecipitation of Dsn1-6His-3FLAG

Yeast cell pulverization and immunoprecipitation was performed as above except that: (1) H_0.15_ buffer was additionally supplemented with 1 mM benzamidine, and one tablet of EDTA-free protease inhibitor tablet (Roche) per every 25 mL. (2) phosphatase inhibitors concentrations were doubled. (3) for elution, 0.5 mg/mL FLAG peptide in lysis buffer was added to beads, gently mixed and incubated at room temperature for 20 min. Beads were concentrated on a magnet, the supernatant removed and snap-frozen in liquid nitrogen before preparation for mass spectrometry as below.

##### Flow cytometry

150 μL of meiotic culture was fixed with 350 μL 96% EtOH and kept at 4°C. Cells were washed with 1 mL 50 mM Tris buffer at pH 7.5, briefly sonicated, spun and resuspended in 500 μL 50mM Tris pH 7.5 before adding 25 μL RNase A (20mg/mL), incubated at 37°C overnight, then spun and washed in 1 mL 50 mM Tris buffer at pH 7.5. Cell pellet was resuspended in 500 μL 50 mM Tris buffer pH 7.5, 10 μL Proteinase K (20mg/mL Amresco) was added and the suspension was incubated at 50°C for 2 h. Sample was washed in 1 mL 50 mM sodium citrate, resuspended in 500 μL sodium citrate and 9.17 μL of 1 mg/mL propidium iodide was added and kept overnight in the dark at 4°C. Sample was sonicated using BioRuptor Twin sonicating device (Diagenode) at LOW setting, 10 min total 30secON/30secOFF. Samples were stored for up to a week in the dark at 4°C and analyzed using on a BD FACS Calibur instrument.

#### Immunofluorescence

Immunofluorescence was used to visualize meiotic spindles. 200 μL meiotic culture was collected, and the pellet resuspended in 3.7% formaldehyde in 0.1 M potassium phosphate at pH 6.4, and fixed overnight at 4°C. Cells were washed 3 times with 1 mL of 0.1 M potassium phosphate buffer pH 6.4 before resuspending in 1 mL of sorbitol-citrate (1.2 M sorbitol, 35 mM citric acid, 0.1 M KH_2_PO_4_). Fixed cells were resuspended in digestion mix (200 μL 1.2 M sorbitol-citrate, 20 μL glusulase (Perkin Elmer) and 6 μL zymolyase (10 mg/mL; AMS Biotechnology (Europe)) for at least 2 h at 30°C, then washed in 1 mL sorbitol-citrate and resuspended in sorbitol-citrate. Spheroplasts were attached to multi-well polylysine-treated slides and fixed in MeOH for 3 min, dried in acetone for 10 s and allowed to dry. Wells were covered with rat anti-tubulin primary antibody (Bio-Rad) at 1:50 dilution in PBS/BSA (1% BSA, 0.04 M K_2_HPO_4_, 0.01 M KH_2_PO_4_, 0.15 M NaCl, 0.1% NaN_3_) for 2 h at room temperature and washed five times in PBS/BSA. Secondary anti-rat FITC conjugated antibody (Jackson Immunoresearch) was added at 1:100 dilution in PBS/BSA, incubated for a further 2 h at room remperature and wells were washed a further five times with PBS/BSA. 3 μL DAPI-MOUNT (1 mg/mL p-phenylenediamine, 0.04M K_2_HPO_4_, 0.01M KH_2_PO_4_, 0.15M NaCl, 0.1% NaN_3_, 0.05 μg/mL DAPI, 90% glycerol) was added to each well and a coverslip placed on the slide before imaging or storing at −20°C.

#### Laser trap experiment

##### Laser trap instrument

The laser trap has been described previously.^70^ Position sensor response was mapped using the piezo stage to raster-scan a stuck bead through the beam, and trap stiffness was calibrated along the two principle axes using the drag force, equipartition, and power spectrum methods. Force feedback was implemented with custom LabView software. During force measurements, bead-trap separation was sampled at 40 kHz while stage position was updated at 50 Hz to maintain the desired tension (force-clamp assay) or ramp-rate (force-ramp assay). Bead and stage position data were decimated to 0.2 kHz before storing to disk.

Bead functionalization and coverslip preparation for laser trap experiments: Native kinetochore particles were linked to beads as previously described.^34^^,^^45^^,^^71^ First, streptavidin-coated polystyrene beads (0.56 μm in diameter, Spherotech, Libertyville IL) were functionalized with biotinylated anti-His_5_ antibodies (QIAGEN, Valencia CA) and stored with continuous rotation at 4°C in BRB80 (80 mM PIPES, 1 mM MgCl_2_, and 1 mM EGTA, pH 6.9) supplemented with 8 mg·mL^-1^ BSA for up to 3 months. Immediately prior to each experiment, beads were decorated with kinetochore particles by incubating 6 pM anti-His_5_ beads for 60 min at 4°C with different amounts of the purified kinetochore material, corresponding to a Dsn1-His-Flag concentration of 7.5 nM. Flow chambers (∼10 μL volume) were made using glass slides, double-stick tape, and KOH-cleaned coverslips, and then functionalized in the following manner. First, 10 - 25 μL of 10 mg/mL biotinylated BSA (Vector Laboratories, Burlingame CA) was introduced and allowed to bind to the glass surface for 15 min at room temperature. The chamber was then washed with 100 μL of BRB80. Next, 75-100 μL of 0.33 mg/mL avidin DN (Vector Laboratories, Burlingame CA) was introduced, incubated for 3 min, and washed out with 100 μL of BRB80. GMPCPP-stabilized biotinylated microtubule seeds were introduced in BRB80, and allowed to bind to the functionalized glass surface for 3 min. The chamber was then washed with 100 μL of growth buffer (BRB80 containing 1 mM GTP and 1 mg/mL κ-casein). Finally, kinetochore particle-coated beads were introduced at an eight-fold dilution from the incubation mix (see above) in a solution of growth buffer containing 1.5 mg/mL purified bovine brain tubulin and an oxygen scavenging system (1 mM DTT, 500 μg/mL glucose oxidase, 60 μg/mL catalase, and 25 mM glucose). The edges of the flow chamber were sealed to prevent evaporation. All laser trap experiments were performed at 23°C.

##### Binding fraction and rupture force measurements

Using the laser trap, individual free beads were placed close to the ends of growing microtubules to allow binding. Binding fraction was defined as the number of free beads that bound a microtubule divided by the total number of free beads tested. Upon binding, the attachments were preloaded with a constant force of ∼3-4 pN. After a brief preload period, during which we verified that the beads were moving at a rate consistent with that of microtubule growth, the laser trap was programmed to ramp the force at a constant rate (0.25 pN s^-1^) until the linkage ruptured, or until the load limit of the trap (∼22 pN) was reached. While < 1% of all trials ended in detachment during the preload period (i.e., before force ramping began), ∼10%–15% reached the trap load limit. These out-of-range events were not included in the distributions or the calculated median rupture forces. In addition to free beads, beads found already attached (i.e., pre-bound) to microtubules were also used for the rupture force measurements (but not for calculating binding fraction). We found no statistically significant difference in the mean rupture force for pre-bound versus free beads that interacted with microtubules and so, we pooled all the data together. Statistics for the data presented in this work are summarized in Table S7.

#### Mass spectrometry

Protein samples were briefly run into an SDS-PAGE gel (NuPAGE Novex 4%–12% Bis-Tris gel, ThermoFisher, UK), in NuPAGE buffer (MES) and visualized using Instant*Blue* stain (Sigma Aldrich, UK). The stained gel areas were excised and de-stained with 50 mM ammonium bicarbonate (Sigma Aldrich, UK) and 100% v/v acetonitrile (Sigma Aldrich, UK) and proteins were digested with trypsin ^72^. In brief, proteins were reduced in 10 mM dithiothreitol (Sigma Aldrich, UK) for 30 min at 37°C and alkylated in 55 mM iodoacetamide (Sigma Aldrich, UK) for 20 min at ambient temperature in the dark. They were then digested overnight at 37°C with 12.5 ng/μL trypsin (Pierce, UK). Following digestion, samples were diluted with an equal volume of 0.1% TFA and spun onto StageTips.^73^ Peptides were eluted in 40 μL of 80% acetonitrile in 0.1% TFA and concentrated down to 1 μL by vacuum centrifugation (Concentrator 5301, Eppendorf, UK). Samples were then prepared for LC-MS/MS analysis by diluting them to 5 μL with 0.1% TFA.

For *CEN-* and *CEN^∗^*-chromatin samples, as well as for the no tag sample in Dsn1-6His-3FLAG immunoprecipitation, LC-MS-analyses were performed on an Orbitrap Fusion Lumos Tribrid Mass Spectrometer (Thermo Fisher Scientific, UK) coupled on-line, to an Ultimate 3000 RSLCnano Systems (Dionex, Thermo Fisher Scientific, UK). Peptides were separated on a 50 cm EASY-Spray column (Thermo Fisher Scientific, UK) assembled in an EASY-Spray source (Thermo Fisher Scientific, UK) and operated at a constant temperature of 50°C. Mobile phase A consisted of 0.1% formic acid in water while mobile phase B consisted of 80% acetonitrile and 0.1% formic acid. Peptides were loaded onto the column at a flow rate of 0.3 μL/min and eluted at a flow rate of 0.2 μL/min according to the following gradient: 2 to 40% buffer B in 150 min, then to 95% in 11 min. Survey scans were performed at 120,000 resolution (scan range 350-1500 m/z) with an ion target of 4E5. MS2 was performed in the Ion trap at rapid scan mode with ion target of 2E4 and HCD fragmentation with normalized collision energy of 27.^74^ The isolation window in the quadrupole was set at 1.4 Thomson. Only ions with charge between 2 and 7 were selected for MS2.

For the Dsn1-6His-3FLAG immunoprecipitation samples (except the no tag sample which was processed as described above), MS-analyses were performed on a Q Exactive mass spectrometer (Thermo Fisher Scientific, UK), coupled on-line to Ultimate 3000 RSLCnano Systems (Dionex, Thermo Fisher Scientific). The analytical column with a self-assembled particle frit^75^ and C18 material (ReproSil-Pur C18-AQ 3 μm; Dr. Maisch, GmbH) was packed into a spray emitter (75-μm ID, 8-μm opening, 300-mm length; New Objective, UK) using an air-pressure pump (Proxeon Biosystems, UK). Mobile phase A consisted of water and 0.1% formic acid; mobile phase B consisted of 80% acetonitrile and 0.1% formic acid. Peptides were loaded onto the column at a flow rate of 0.5 μL/min and eluted at a flow rate of 0.2 μL/min according to the following gradient: 2 to 40% buffer B in 180 min, then to 95% in 16 min.

The resolution for the MS1 scans was set to 70,000 and the top 10 most abundant peaks with charge ≥ 2 and isolation window of 2.0 Thomson were selected and fragmented by higher-energy collisional dissociation^74^ with normalized collision energy of 30. The maximum ion injection time for the MS and MS2 scans was set to 20 and 60 ms respectively and the AGC target was set to 1E6 for the MS scan and to 5E4 for the MS2 scan. Dynamic exclusion was set to 60 s.

The MaxQuant software platform version 1.6.1.0 (released in April 2018) was used to process raw files and searches were conducted against the *Saccharomyces cerevisiae* (strain SK1) complete/reference proteome set of the *Saccharomyces* Genome Database (released in May, 2019), using the Andromeda search engine.^76^ The first search peptide tolerance was set to 20 ppm, while the main search peptide tolerance was set to 4.5 pm. Isotope mass tolerance was set to 2 ppm and maximum charge to 7. Maximum of two missed cleavages were allowed. Carbamidomethylation of cysteine was set as fixed modification. Oxidation of methionine and acetylation of the N-terminal as well as phosphorylation of serine, threonine and tyrosine were set as variable modifications. LFQMS analysis was performed by employing the MaxLFQ algorithm).^77^ For peptide and protein identifications FDR was set to 1%.

#### Quantitative analysis of mass spectrometry data

LFQMS data was processed using Bioconductor *DEP* R package.^78^ Briefly, proteins with indicators Reverse “+” and Potential.contaminant “+” were removed from the dataset. Data were filtered to only keep proteins detected in all replicates of at least one condition, LFQ intensities were log_2_-transformed and normalized using variance-stabilized normalization. Then, imputation was performed using “MinProb” function, with q = 0.001. Log_2_(Fold Change) and p values were calculated using linear models and empirical Bayes method.

Pie charts in Figure 1B were generated by identifying proteins enriched over no tag control with p value < 0.01 and Log_2_(Fold Change) > 4. A single no tag sample was used for the kinetochore sample. Three previously published metaphase I samples were used as no tag control for *CEN* and *CEN*^∗^ chromatin samples (PRIDE identifier: PXD012627, samples Sgo1_no_tag_1-3^37^)).

Heatmaps in Figures 1, 2A, and S1 were generated using *DEP* package plot_heatmap() function with a modified color scheme. To generate the heatmap shown in Figure 2, the *CEN/CEN*^∗^ ratio was determined for each protein in each condition. Data were filtered to reject proteins that failed to show *CEN/CEN*^∗^ ratio > |2| and p value < 0.05, which we defined as significant enrichment, in at least one condition.

Cumulative plots shown in Figure 4A were generated using DEP-processed data. Following filtering, normalization and imputation, the log_2_-transformed data were exponentiated to obtain LFQ intensities. These were then summed for individual complexes in each condition, log_2_-transformed and ratios between conditions were determined and plotted. Complexes were defined as follows: Cbf3 complex: Skp1, Cbf2, Cep3, Ctf13; Cse4, Mif2: Cse4, Mif2; Ctf19 complex: Ame1, Okp1, Chl4, Nkp1, Mcm22, Mcm16, Nkp2, Ctf3, Ctf19, Wip1, Cnn1 (note Iml3 and Mcm21 were excluded, because *IML3* and *MCM2*1 are deleted in some of the samples); Mtw1 complex: Mtw1, Nnf1, Nsl1, Dsn1; Outer KT: Spc105, Kre28, Dad3, Dad1, Dad4, Spc19, Duo1, Dam1, Ask1, Hsk3, Spc34, Dad2, Ndc80, Nuf2, Spc24, Spc25; SPBs and MTs: Spc72, Spc110, Spc42, Cnm67, Spo21, Ady3, Nud1, Mpc54, Don1, Mps2, Spc97, Spc98, Tub2, Stu1, Stu2, Tub3, Tub1, Bik1, Cin8, Ase1, Tub4; cohesin: Smc1, Smc3, Irr1, Rad61, Pds5, Rec8; Msh4/Msh5: Msh4, Msh5; SZZ: Spo16, Spo22, Zip2.

The LFQMS data used in Figures 1, 2, S1, and S2 utilizes both haploid and diploid cycling cells samples, and, following initial analyses, some diploid samples were rejected from the original dataset. For LFQMS data shown in all other figures, only haploid cycling cells were rejected, as no haploid samples were obtained for *iml3Δ* and *mcm21Δ* cells. Each sample was injected only once (no technical replicates). Number of biological replicates analyzed in kinetochore proteomics: n = 3 for all conditions apart from n = 1 for no tag. Number of biological replicates in *CEN* and *CEN^∗^* proteomics: Figures 1, 2, and S2: *CEN*, cycling cells, n = 5; *CEN^∗^*, cycling cells, n = 6; *CEN*, prophase I, n = 4; *CEN^∗^*, prophase I, n = 3, *CEN* and *CEN^∗^*, metaphase I, n = 3. Figures 4 and S4: wild type: *CEN* and *CEN^∗^*, cycling cells, n = 6; *CEN* and *CEN^∗^*, prophase I, n = 4 and 3, respectively; CEN and CEN^∗^, metaphase I, n = 3. *iml3Δ: CEN*, cycling cells, n = 6; *CEN*, prophase I, n = 4; *CEN*, metaphase I, n = 3. *mcm21Δ: CEN*, cycling cells, n = 3; *CEN*, prophase I, n = 3; *CEN*, metaphase I, n = 3.

#### Chromatin immunoprecipitation

Cells in 50 mL of SPO culture at OD_600_ ≥ 1.9, or 100 mL YPDA culture at OD = 0.8 were fixed by addition of formaldehyde to 1%. Following 2 h crosslinking, cultures were spun, supernatant was removed and the pellet washed twice in 10 mL of ice-cold TBS (20 mM Tris/HCl at pH 7.5, 150 mM NaCl) and once in 1 mL of ice-cold FA lysis buffer (50 mM HEPES-KOH at pH 7.5, 150 mM NaCl, 1 mM EDTA, 1% v/v Triton X-100, 0.1% w/v Sodium Deoxycholate) with 0.1% w/v SDS and the snap-frozen pellet was kept at −80°C. Next, the pellet was resuspended in 0.4 mL of ice-cold FA buffer supplemented with EDTA-free protease inhibitors (Roche), 1 mM PMSF (cFA) and 0.5% w/v SDS. Cells were lysed using silica beads (Biospec Products) in a Fastprep Bio-pulverizer FP120, with two 30 s rounds of bead-beating at maximum power, with intervening 10 min incubation on ice. The lysate was collected and spun for 15 min at 14 krpm, supernatant was rejected, and the pellet was washed with 1 mL of cFA supplemented with 0.1% w/v SDS. Following another spin for 15 min at 14 krpm, the pellet was resuspended in 0.5 mL of cFA supplemented with 0.1% w/v SDS, and sonicated using BioRuptor Twin sonicating device (Diagenode) at HIGH setting, 30 × 30 s at 4°C. Extract was spun for 15 min at 14 krpm, supernatant recovered, an additional 0.5 mL of cFA supplemented with 0.1% w/v SDS was added, and the mixture was spun again for 15 min at 14 krpm. 1 mL of supernatant was then added to a fresh tube containing 0.3 mL of cFA supplemented with 0.1% w/v SDS. From this solution, 1 mL was used for the IP and 100 μL was stored at −20°C as input sample. Protein G Dynabeads (Invitrogen) were washed four times in 1 mL of ice-cold cFA lysis buffer with 0.1% w/v SDS. For the IP, 15 μL of pre-washed Dynabeads as well as the appropriate amount of antibody (mouse anti-Ha (12CA5, Roche, 7.5 μL), mouse anti-Flag (M2, Sigma, 5 μL)) were added to 1 mL of lysate and incubated overnight at 4°C. Next, the supernatant was removed and the Dynabeads were incubated in 1 mL of ChIP wash buffer 1 (0.1% w/v SDS, 275 mM NaCl, FA) with rotational mixing for 5 min at room temperature. This washing was repeated with ChIP wash buffer 2 (0.1% SDS, 500 mM NaCl, FA), ChIP wash buffer 3 (10 mM Tris/HCl at pH 8, 0.25 M LiCl, 1 mM EDTA, 0.5% v/v NP-40, 0.5% w/v Sodium Deoxycholate), and TE (10 mM Tris/HCl at pH 8, 1 mM EDTA). 200 μL of 10% w/v Chelex (Biorad) suspension in DEPC-treated sterile water (VWR) was added to the Dynabeads as well as to 10 μL of the thawed input sample. This was incubated for 10 min at 100°C, cooled, and 2.5 μL of 10 mg/mL Proteinase K (Promega) was added. Incubation at 55°C for 30 min was followed by an incubation for 10 min at 100°C and cooling samples on ice. Samples were spun and 120 μL of supernatant of both IP and input samples was collected. qPCR was performed as described in Verzijlbergen et al.^79^ Mean values are shown from a minimum of 3 biological repeats, with error bars representing standard error. Primers for qPCR analysis are listed in Table S6.

#### Western blotting

For western immunoblotting, samples were fixed in trichloroacetic acid for 10 min, acetone-washed and whole cell extracts prepared by bead-beating in TE-containing protease inhibitors before transferring to nitrocellulose membrane. Antibodies used were rabbit anti-Pgk1 (lab stock,1:50000), mouse anti-Myc (Covance/Biolegend 9E10, 1:1000), mouse anti-Ha (Mono HA.11, Covance, 1:1000), sheep anti-mouse-HRP (GE Healthcare, 1:5000), donkey anti-rabbit-HRP (GE Healthcare, 1:10000).

#### Sporulation and viability assays

Viability of mitotically cycling cells was determined by growing the cells to OD_600_ = 1, and diluting them 1000 times, then plating 400 μL of cell suspension onto YPDA plates. Viability of meiotic cells was determined by dissecting 36 or more tetrads of a homozygous diploid carrying the mutation of interest. Viability drop following return to growth was determined by growing cells as described in “Meiotic induction” up until cells were moved into SPO medium. Then, for each SPO culture, 300 cells were plated at t = 0 h and t = 5 h. Cells were counted two days after and ratio 5 h/0 h was determined. Viability of spores using random spore analysis was determined by growing cells as described in “Meiotic induction” and then incubating in SPO medium for 48 h at 30°C. Sporulation efficiency was determined by light microscopy. 1 mL of miotic culture was then spun, resuspended in 100 μL of zymolyase solution and incubated for 2 h at 30°C. The mixture was spun again and resuspended in 600 μL of 1.67% NP-40, vortexed at high speed for 10 min and spun. Pelleted spores were resuspended in 500 μL of water and sonicated using BioRuptor Twin sonicating device (Diagenode) at HIGH setting, 2 × 30 s at 4°C. Tetrad disruption was confirmed under light microscope and, if intact tetrads were observed, the spore suspension was vortexed for another 10 min or until only individual spores were seen. Spores were diluted in water and equal spore numbers were plated (hemocytometer was used to determine spore concentration).

#### Chromosome segregation assay

Diploid strains with either one copy (heterozygous) or both copies (homozygous) of chromosome V marked with GFP were induced to sporulate at 30°C. To score GFP dots, cells were fixed as previously described^18^ and for each biological repeat 100 tetranucleate cells were counted at 8 or 10 h after transfer to sporulation medium.

#### Live-cell imaging

##### Equipment

Live-cell imaging shown and analyzed in Figures 3B, 3C, 4D–4G, 5C, 6A, 7C–7G, S3E–S3G, S5B–S5E, S6A, and S7B–S7E was performed at 30°C on a Zeiss Axio Observer Z1 (Zeiss UK, Cambridge) equipped with a Hamamatsu Flash 4 sCMOS camera, Prior motorised stage and Zen 2.3 acquisition software. Live-cell imaging shown in Figures S7A, 5A, and 5B (both at 25°C) used spinning-disk confocal microscopy employing a Nikon TE2000 inverted microscope with a Nikon X100/1.45 NA PlanApo objective, attached to a modified Yokogawa CSU-10 unit (Visitech) and an iXon° Du888 EMCCD camera (Andor), controlled by Metamorph software (Molecular Devices).

##### Imaging chamber preparation

Cells were imaged at the indicated temperature in 4-well or 8-well Ibidi glass-bottom dishes coated with concanavalin A. For all imaging experiments apart from that shown in Figures 7E, 7F, and S7E, cells were grown as described in the “Meiotic induction” section to achieve rapid and synchronous entry into meiosis. Such obtained meiotic cultures were pre-grown in SPO-containing culture flasks for ∼3 h (*pCLB2-CDC20* and asynchronous), 4.5 h (inducible-*NDT80*) or 1 h (*pCUP1:IME1 pCUP1:IME4)* before transfer to Ibidi dishes, where they were left to attach, while in SPO, for 20 – 30 min. For prophase I and pre S-phase block-release experiments, beta-estradiol and copper (II) sulfate, respectively, was added immediately before the first image was acquired. Imaging began about 30 min after attachment was completed, with images being acquired every 7.5 – 15 min for 10 – 12 h. For the experiment shown in Figure S7E, where rapid entry into meiosis upon exposure to SPO was undesirable, diploids were cultured in YPDA at OD < 1 before transfer to Ibidi dishes, and they were left to attach, while in YPDA, for 20 – 30 min. SPO medium was added to the imaging chamber directly (< 3 min) before imaging commenced.

##### Image acquisition and analysis

8 – 11 z sections were acquired with 0.6 – 0.8 μm spacing. Images were analyzed using ImageJ (National Institutes of Health). Final image assembly was carried out using Adobe Photoshop and Adobe Illustrator. Where signal intensity was measured (Figures 3C, 4D, 4F, 7C, 7E, and S7E), a circular region was drawn that encompassed the region of interest (ROI), and mean ROI intensity was measured. The same size region was then drawn in an area in the vicinity, and the mean intensity of this area was measured and defined as background intensity (bROI). The signal presented in figures is the mean ROI signal minus mean bROI signal. Spindle length of metaphase I-arrested cells in Figure 6A was measured at its maximal length observed in the time-lapse. Cell cycle delay quantified in Figure S6A was measured by calculating time between the first time point in which a bilobed Mtw1-tdTomato signal was observed and the first time point in which Mtw1-tdTomato foci reach opposite ends of the cell.

### Quantification and Statistical Analysis

R software was used for statistical analysis. Statistical details can be found in figure legends, apart from details about analysis of LFQMS data presented in Figures 1C, 2A–2D, 4A, S1, S2, and S4, which are described in “Quantitative analysis of mass spectrometry data” part of the Methods section. Details about imaging quantification can be found in “Image acquisition and analysis” part of the Methods section.

## Supplemental Information

Document S1. Figures S1–S7 and Tables S5–S7

Table S1. Protein Groups from Clustering Analysis of CEN and CEN∗ Chromatin, Related to Figure 1

Table S2. Protein Groups from Clustering Analysis of the Kinetochore Datasets, Related to Figure 2

Table S3. Protein Groups after Clustering Analysis of CEN/CEN∗ Chromatin, Related to Figure 2

Table S4. Yeast Strains Used in This Study, Related to STAR Methods

Document S2. Article plus Supplemental Information
