# Supplementary material for: The Proteomic Landscape of Centromeric Chromatin Reveals an Essential Role for the Ctf19CCAN Complex in Meiotic Kinetochore Assembly
Source: Curr Biol. 2021 Jan 25;31(2):283–296.e7. doi: 10.1016/j.cub.2020.10.025 (PMC7846277; doi:10.1016/j.cub.2020.10.025)
Supplement: Document S1. Figures S1–S7 and Tables S5–S7 [file mmc1.pdf]

Current Biology, Volume *31*

Supplemental Information

The Proteomic Landscape of Centromeric Chromatin Reveals an Essential Role for the Ctf19^{CCAN} Complex in Meiotic Kinetochore Assembly

Weronika E. Borek, Nadine Vincenten, Eris Duro, Vasso Makrantoni, Christos Spanos, Krishna K. Sarangapani, Flavia de Lima Alves, David A. Kelly, Charles L. Asbury, Juri Rappsilber, and Adele L. Marston

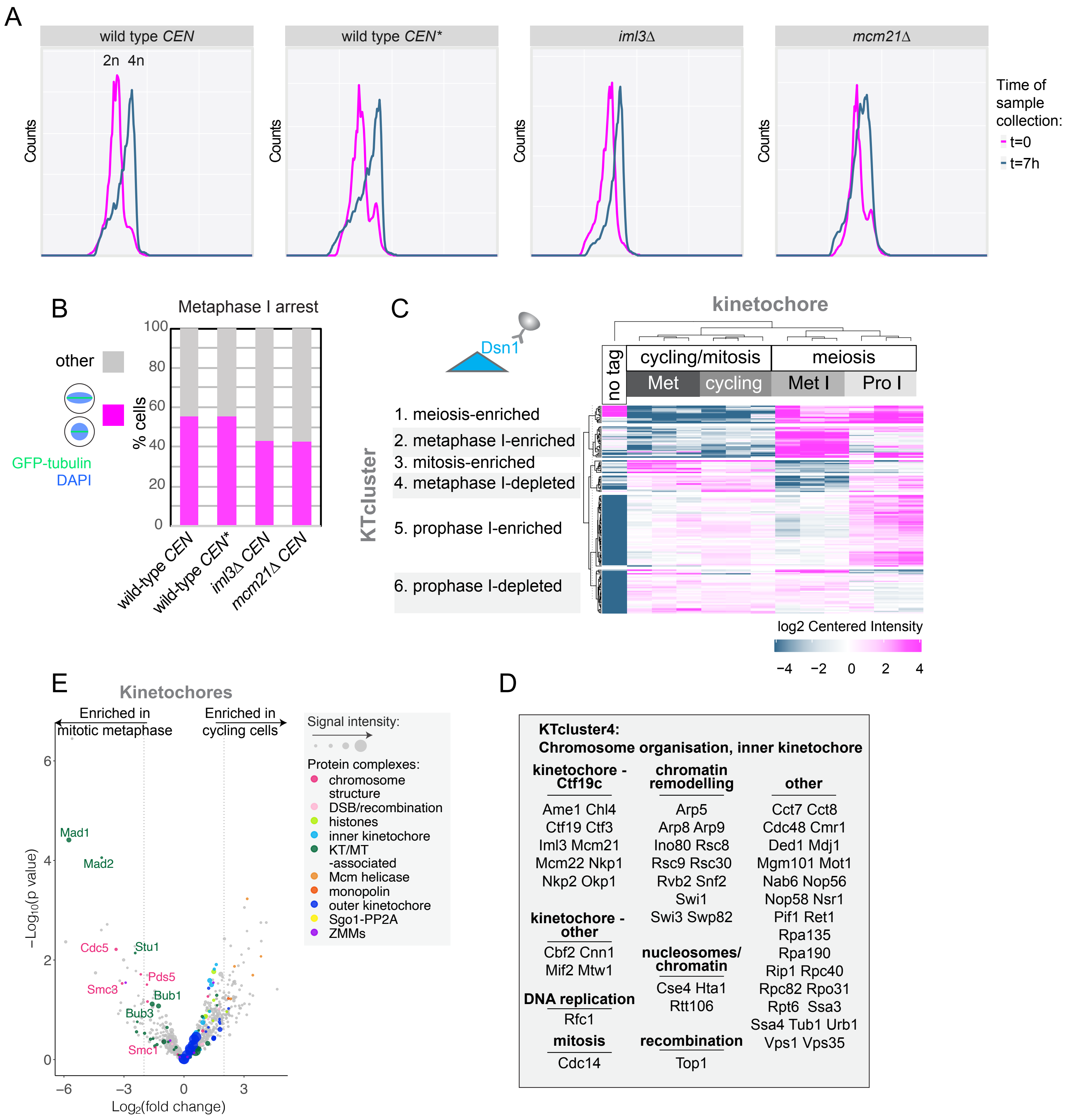

Figure S1. Comparison of mitotic and meiotic kinetochore proteomes. Related to Figure 2. (A) Flow cytometry profiles of representative of prophase I-arrested wild-type and Ctf19c-deficient cells used for purification of *CEN* and *CEN** chromatin. Note that although DNA replication is not complete in *mcm21*Δ cells due to consistently delayed meiotic progression, the majority of cells must have entered meiosis because their kinetochores undergo a dramatic change and the outer kinetochore is lost (Figure 4A). (B) Immunofluorescence analysis of metaphase I arrested cells used for purification of *CEN* and *CEN** chromatin. (C) Kinetochore composition varies across cell cycle stages. Clustering analysis of kinetochore samples (k-means). A cut-off of Log_2(fold change) > 1 and p < 0.05 was used. (D) Proteins shown in KTcluster4 containing prophase I-enriched proteins in (C) are listed. (E) Kinetochore-associated proteome of cycling cells is similar to that of mitotic metaphase-arrested cells. Volcano plot showing LFQMS-identified proteins co-purifying with Dsn1-6His-3FLAG in cycling cells vs. mitotic metaphase-arrested (benomyl) cells. Log_2(fold change) between conditions are shown with their corresponding p values (see methods). Dashed line indicates $|Log_2$(fold change)$| = 2$. See also Table S2.

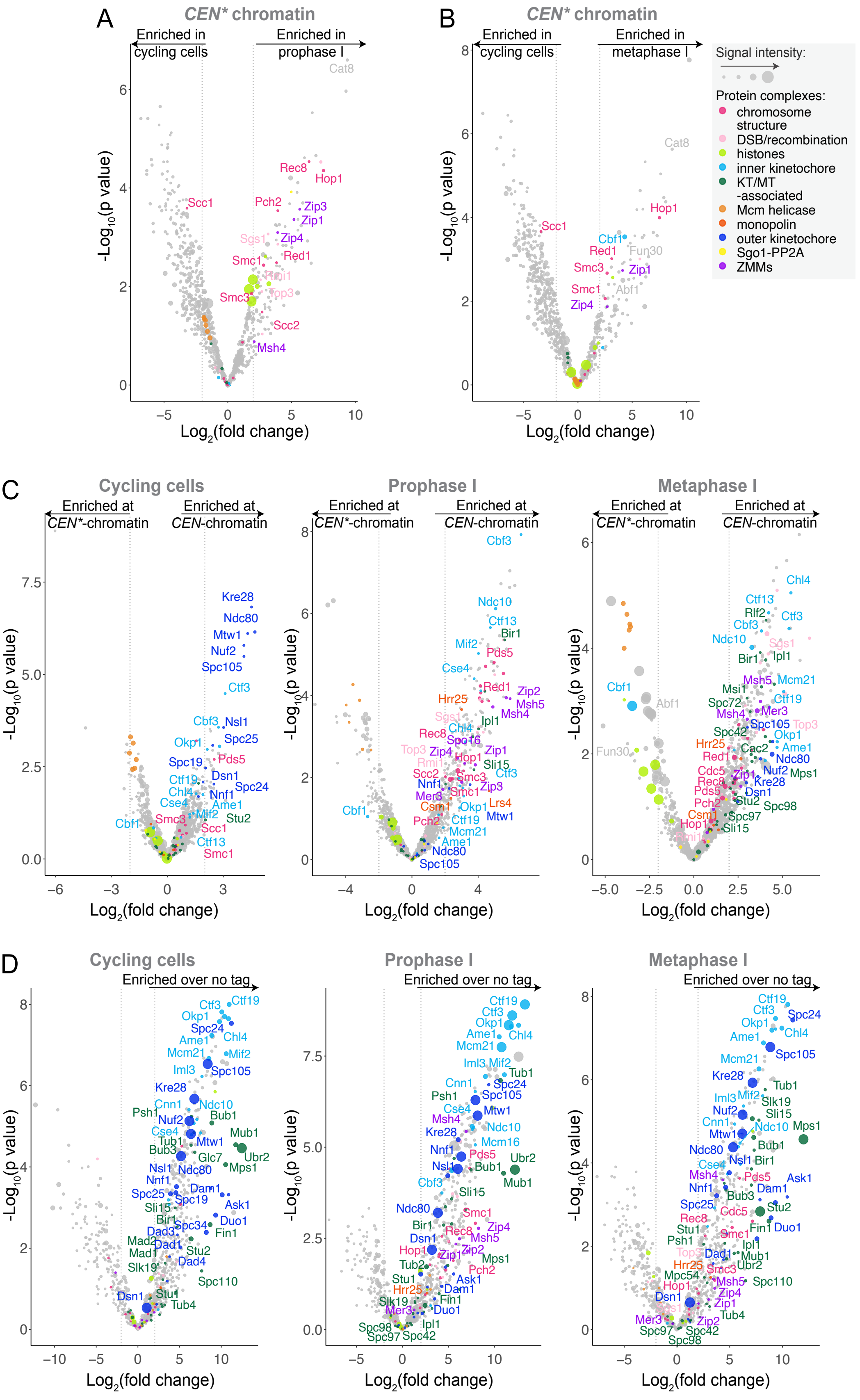

Figure S2. Overview of protein enrichment in *CEN* and kinetochore purifications from cycling, prophase I and metaphase I cells. Related to Figure 2.

(A and B) Comparison of *CEN** chromatin isolated from cycling cells and two meiotic stages. Volcano plots showing LFQMS-identified proteins co-purifying with *CEN** plasmids immunopurified from cells arrested in prophase (*ndt80*Δ, A) and metaphase I (*pCLB2-CDC20*, B) as compared to cycling cells. Log_2(fold change) between conditions are presented with their corresponding p values (see methods). Dashed line indicates $|Log_2(\text{fold change})| = 2$. Legend to right is for all panels in this figure. (C and D) *CEN* chromatin (C) and kinetochore (D) composition varies depending on cell cycle stage. (C) Volcano plots showing LFQMS-identified proteins co-purifying with *CEN* and *CEN** plasmids immunopurified from cells that are cycling, arrested in prophase (*ndt80*Δ) and metaphase I (*pCLB2-CDC20*). (D) Volcano plot showing LFQMS-identified proteins co-purifying with Dsn1-6His-3FLAG immunopurified from cells that are cycling, arrested in prophase (inducible-*NDT80*) and metaphase I (*pCLB2-CDC20*).

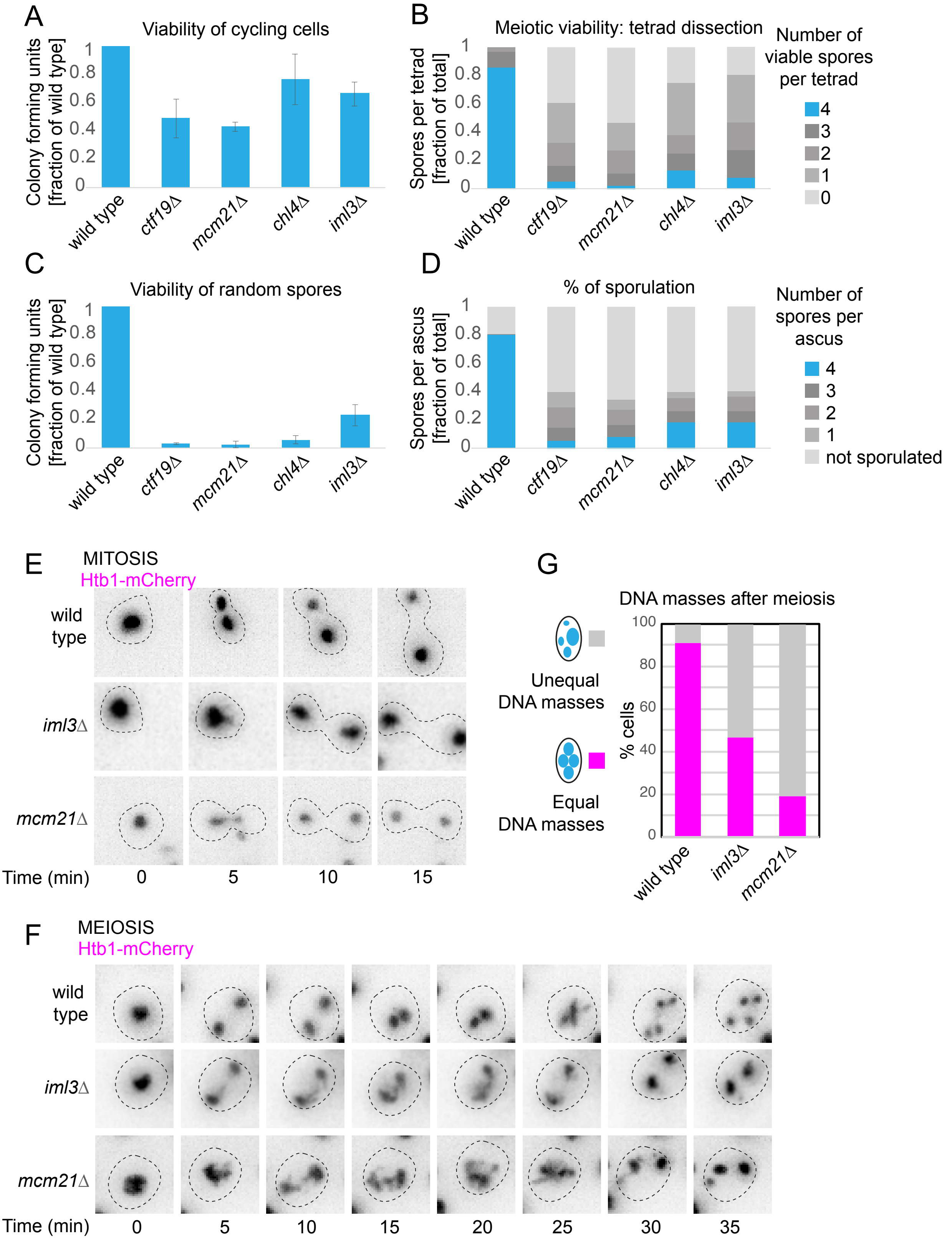

Figure S3. Ctf19c^{CCAN} becomes essential in meiosis. Related to Figure 3.
(A) Deletion of non-essential Ctf19c^{CCAN} components mildly impairs mitotic viability. Viability of cycling cells of the indicated genotypes is shown after plating as proportion of wild type. n = 3 – 4 biological replicates, minimum of 200 cells plated for each genotype. (B-D) Meiotic viability of Ctf19c^{CCAN}-deficient cells is reduced compared to wild-type cells. (B) Viability of spores in four-spore tetrads. The number of viable progeny was scored (n = 36 tetrads). (C) Random spore viability. n = 2 biological replicates. (D) Sporulation efficiency. n = 2 biological replicates. (E-G) Deletion of *IML3* and *MCM21* causes gross chromosome missegregation in meiotic, but not mitotic, cells. (E) Mitotically cycling wild-type, *iml3Δ* and *mcm21Δ* cells expressing Htb1-mCherry were imaged. t = 0 min time-point is defined as the last time-point before DNA masses start to separate. (F) Wild-type, *iml3Δ* and *mcm21Δ* cells expressing Htb1-mCherry were released from prophase I arrest and imaged through meiosis. t = 0 min time-point is defined as the last time-point before DNA masses start to separate. (G) Quantification of F. 58-92 cells were scored.

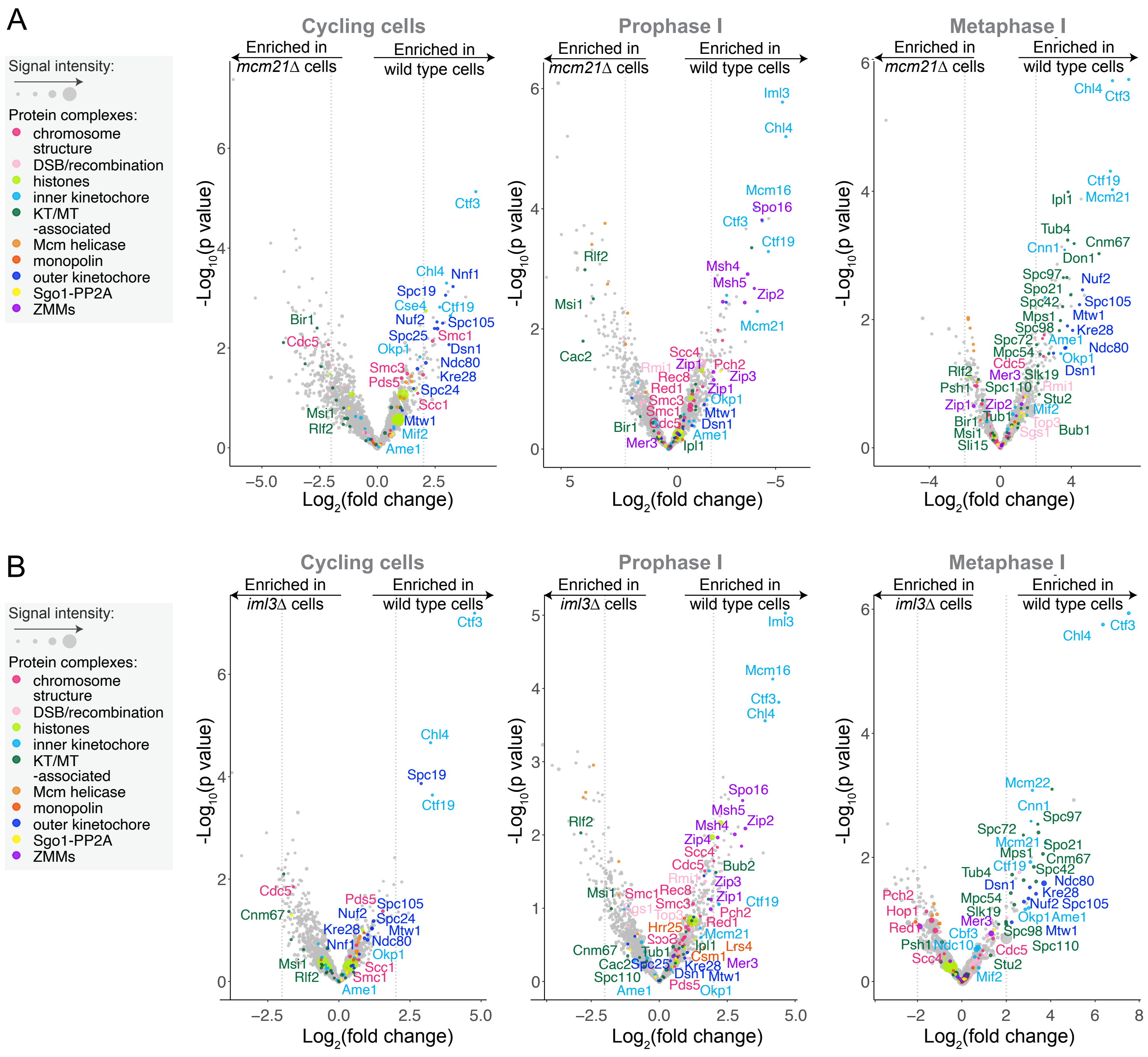

Figure S4. Kinetochores fail to assemble in meiosis in *iml3Δ* and *mcm21Δ* cells. Related to Figure 4. (A and B) The Ctf19c^{CCAN} plays a central role in kinetochore composition during meiosis. (A) Volcano plots showing LFQMS-identified proteins co-purifying with *CEN* chromatin in wild-type vs. *mcm21Δ* cells from mitotically cycling cells, meiotic prophase I and meiotic metaphase I cells. Log_2(fold change) between conditions are presented with their corresponding p values (see methods). Dashed line indicates $|Log_2$(fold change)$| = 2$. Plots show data for cycling cells, cells arrested in prophase I (*ndt80Δ*) allele and cells arrested in metaphase I (*pCLB2-CDC20*). (B). Volcano plots showing the LFQMS-identified proteins co-purifying with *CEN* chromatin in wild-type vs. *iml3Δ* cells at different cell cycle stages. Log_2(fold change) between conditions are presented with their corresponding p values (see methods). Dashed line indicates $|Log_2$(fold change)$| = 2$. Plots show data for cycling cells, cells arrested in prophase I (*ndt80Δ*) and cells arrested in metaphase I (*pCLB2-CDC20*).

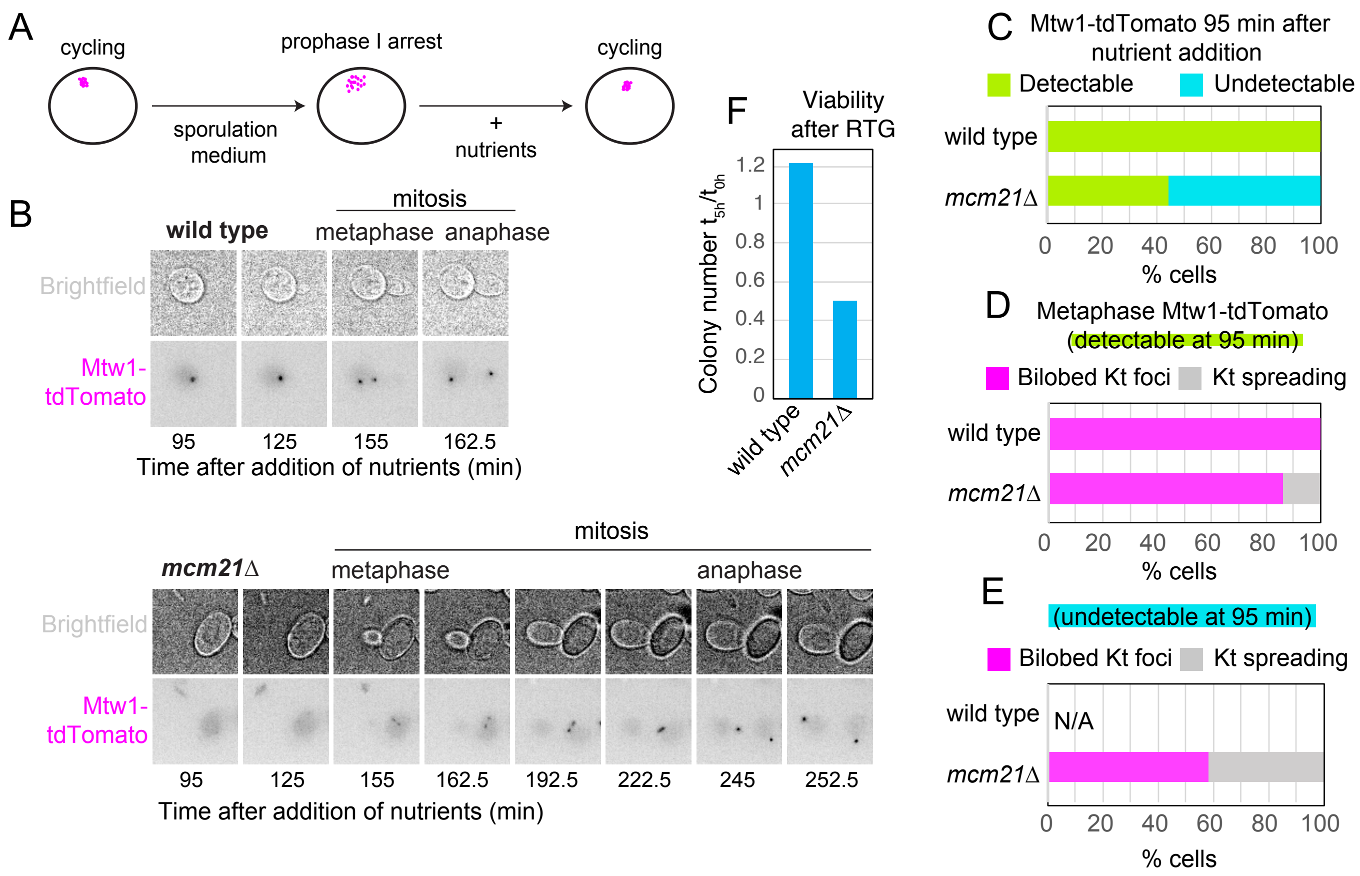

Figure S5. Defective kinetochores persist in Ctf19c^{CCAN} deletion mutants after return to growth. Related to Figure 5.

(A-E) Mtw1c^{MIS12c} binding and kinetochore function is not restored in *iml3*Δ and *mcm21*Δ cells in the mitotic division following return to growth. (A) Schematic of return-to-growth experiments. (B) Mtw1-tdTomato fails to localise to kinetochores in the absence of *MCM21* following return to growth. Nutrients were added to wild-type and *mcm21*Δ cells arrested in prophase I. Imaging commenced 95 minutes after addition of nutrients. (C-E) Loss of kinetochore-localised Mtw1-tdTomato correlates with further kinetochore spreading. (C) Fraction of cells with detectable (green) and undetectable (blue) Mtw1-tdTomato signal 95 min after return to growth is shown. n > 64 cells. (D and E) Following the cells scored in (C), the appearance of Mtw1-tdTomato signal at metaphase in those cells in which foci were detectable (n > 28 cells, D) or undetectable (n > 29 cells, E) upon return to growth is shown. Numbers of cells in C vs. D and E are not identical, as it was not possible to score all the cells later in mitosis. (F) Cells lacking *IML3* or *MCM21* exhibit loss of viability following return to growth. Wild-type, *iml3*Δ and *mcm21*Δ cells were induced to sporulate and plated at t0h (before meiosis) and t5h (prophase I arrest). Viability drop from t0h to t5h was calculated. n > 158 cells. RTG – return to growth.

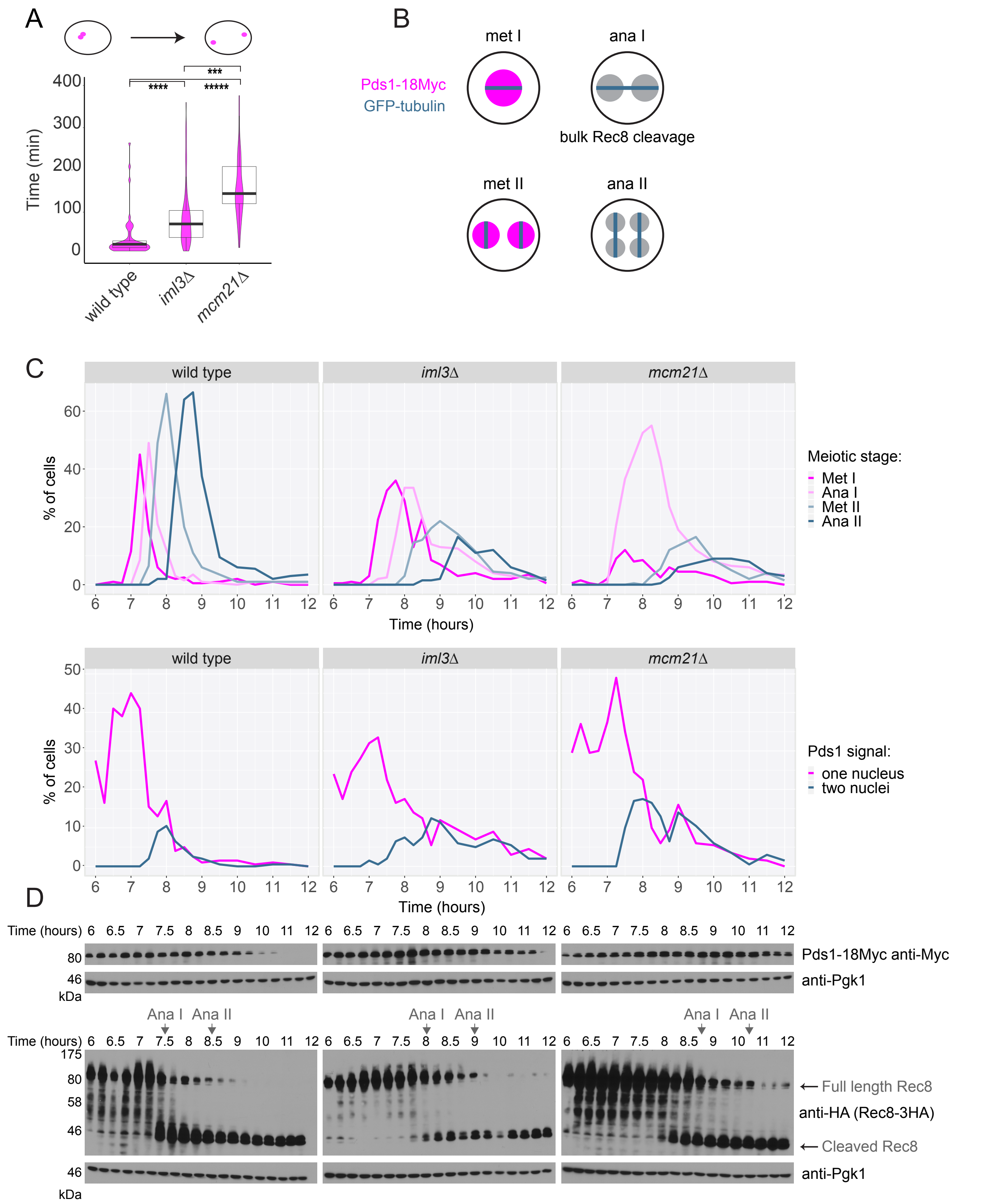

Figure S6. Ctf19c^{CCAN} deletion mutants show a metaphase I delay. Related to Figure 6.

(A) Cells lacking *IML3* and *MCM21* show delayed cell cycle progression. Asynchronously growing wild-type, *iml3*Δ and *mcm21*Δ cells expressing Mtw1-tdTomato and Ndc80-GFP were imaged. Time between emergence of bilobed kinetochore structure and anaphase (when two Mtw1-tdTomato foci reach opposite ends of mother and daughter cells) was measured. ***p < 10-4, ****p < 10-8, *****p < 10-15; Mann-Whitney test. n > 30 cells. Whiskers represent 1.5 IQR, the middle line is median, the box encompasses two middle quartiles of the data. (B-D) Cells lacking *IML3* and *MCM21* are delayed in formation of meiosis II spindles, cleavage of cohesin (Rec8^{REC8}) and degradation of securin (Pds1^{SECURIN}). (B) Schematic of the experiment shown in C and D. The expected spindle phenotypes, the presence or absence of Pds1^{SECURIN}, and the presence or absence of cleaved Rec8^{REC8} are indicated for the different cell cycle stages. (C and D) Wild-type, *iml3*Δ and *mcm21*Δ cells were synchronously released from prophase I and samples were collected at indicated times. (C) Spindle morphology and the presence of Pds1^{SECURIN} were scored by immunofluorescence. (D) Anti-Myc (Pds1-18Myc), anti-HA (Rec8-3HA) and anti-Pgk1 (loading control) immunoblots. Arrows (Ana I and Ana II) represent the onset of anaphase I and anaphase II, based on Rec8 cleavage, respectively.

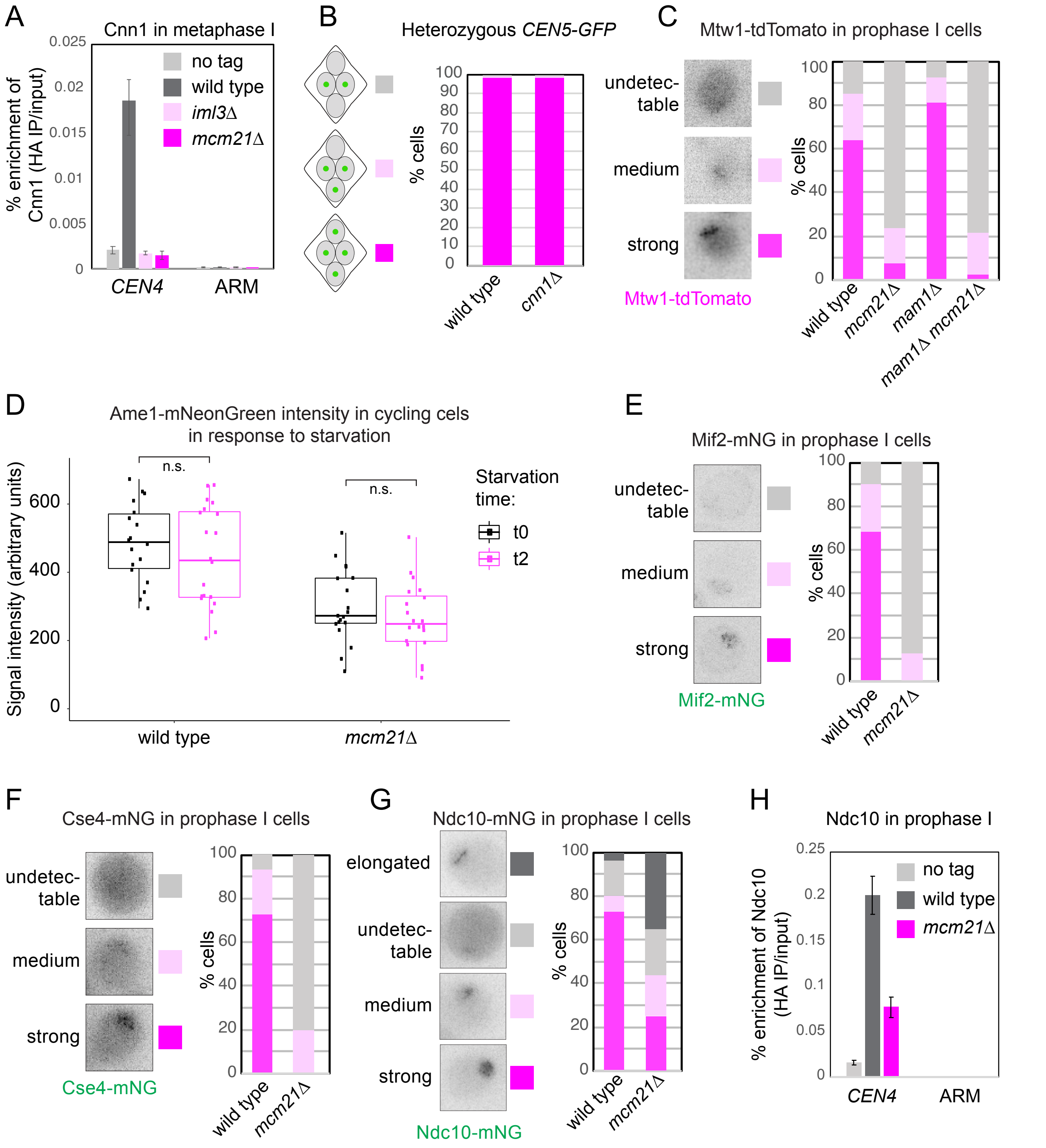

Figure S7. Inner kinetochore proteins fail to localise to meiotic kinetochores in the absence of non-essential Ctf19c^{CCAN} components. Related to Figure 7.

(A) Cnn1$^{CENP\text{-}T}$ is lost from kinetochores in the absence of *IML3* and *MCM21*. Metaphase I-arrested wild-type, *iml3Δ* and *mcm21Δ* cells carrying *pCLB2-CDC20* and *CNN1-6HA* together with untagged control were subjected to anti-HA ChIP-qPCR. Error bars represent standard error (n = 3 biological replicates). $p < 0.05$, paired t-test. (B) *cnn1Δ* cells segregate chromosomes faithfully in meiosis. Wild-type and *mcm21Δ* cells with both copies of chromosome V marked with GFP were sporulated. The percentage of tetra-nucleate cells with the indicated patterns of GFP dot segregation was determined. n = 100 tetrads. (C) Loss of kinetochore integrity in *mcm21Δ* cells is not rescued by deletion of *MAM1*. Strains of the indicated genotypes were imaged immediately after release from prophase I arrest and Mtw1-tdTomato appearance was scored (n > 48 cells). (D) Cycling wild-type and *mcm21Δ* cells expressing Ame1-mNeonGreen were imaged immediately after moving to SPO medium and after 2 hours. Data for t0 (shown in black) is identical to that in Figure 7E as the experiment was performed at the same time. Whiskers represent 1.5 IQR, the middle line is median, the box encompasses two middle quartiles of the data. (E-H) Loss of non-Ctf19c^{CCAN} inner kinetochore proteins Mif2$^{CENP\text{-}C}$ (E), the centromeric nucleosome Cse4$^{CENP\text{-}A}$ (F) and Ndc10 (G and H) in meiotic prophase cells lacking *MCM21*. (E-G) Wild-type and *mcm21Δ* cells were imaged immediately after release from prophase I arrest and Mif2-mNeonGreen (E, n > 56 cells), Cse4-mNeonGreen (F, n > 58 cells) or Ndc10-mNeonGreen (G, n > 60 cells) was scored. (H) Prophase I-arrested wild-type and *mcm21Δ* cells carrying *NDC10-6HA* together with untagged control were subjected to anti-HA ChIP-qPCR. Error bars represent standard error (n = 4 biological replicates). $p < 0.05$, paired t-test.

Plasmid	Name	Description	Source
AM747	*LacI-3FLAG:URA3*	pSB737 from Biggins lab, LacI-*3FLAG:URA3* integrates at *URA3* locus following StuI digestion	[S1]
AM1103	<*CEN3-TALO8*>*:TRP*	pSB964 from Biggins lab, *CEN3* minichromosome	(S1]
AM1106	<*CEN3*-TALO8*>*:TRP*	pSB972 from Biggins lab, CCG -> GCT mutation introduced in CDEIII, *CEN3** minichromosome	[S1]
AM1278	pWS082	sgRNA entry vector	[S2]
AM1279	pWS158	STRONG Cas9 - gRNA gap repair expression vector for budding yeast CRISPR, *URA3*	[S2]
AM1295	pWS082_*CSE4*gRNA.	sgRNA entry vector with sgRNA guide for *CSE4*	this study
AM1362	pLC605-*NDC80-3v5-del2-88*	p888 from Unal lab. 3v5 C-terminal tagged Ndc80 without the first 2-28 residues	[S3]
AM1604	pFA6a-*mNeonGreen-KlLEU2*	mNeonGreen tagging plasmid with *Kluyveromyces lactis LEU* marker	this study
AM1467	pWS082_*NDC80*_gRNA	sgRNA entry vector with sgRNA guide for *NDC80*	this study

Table S5. Plasmids used in this study. Related to STAR methods.

primer	sequence	description
AMo782	AGATGAAACTCAGGCTACCA	qPCR Forward Primer, chromosome IV arm
AMo783	TGCAACATCGTTAGTTCTTG	qPCR Reverse Primer, chromosome IV arm
AMo794	CCGAGGCTTTCATAGCTTA	qPCR Forward Primer, chromosome IV centromere
AMo795	ACCGGAAGGAAGAATAAGAA	qPCR Reverse Primer, chromosome IV centromere
AMo6663	CAACGATGTGCTTCAGTATTAC	Forward primer to amplify sgRNA from PWS082 derivatives
AMo6664	GCTGTAGATATCCTGCACTC	Reverse primer to amplify sgRNA from PWS082 derivatives
AMo6723	CTGCGTTTATACGTCTCAGTTTTAGAGC	Forward primer to amplify Ca9 vector backbone for CRISPR transformations
AMo6724	GTTTCACTTTCCGTCTCAAGTC	Reverse primer to amplify Ca9 vector backbone for CRISPR transformations
AMo6819	GAATGCTGGTCGCTATACTGCTATCTTCCGTTGGCGCAAAC	Forward primer ~1kb upstream of Ndc80 ORF start
AMo6846	GACTTTCGATTTCTAGATTACCTGCT	Forward primer to generate sgRNA to internally tag Cse4
AMo6847	AAACAGCAGGTAATCTAGAAATCG	Reverse primer to generate sgRNA to internally tag Cse4
AMo6853	CGTTCGTTCTCCTGCTTAGAGAGC	reverse internal primer to amplify *NDC80* ORF
AMo7441	AAACTGTGATGTAGCACATGTTGAAA	Reverse primer to generate sgRNA to truncate Ndc80
AMo7442	GACTTTTCAACATGTGCTACATCACA	Forward primer to generate sgRNA to truncate Ndc80
AMo8660	CGACAAAGAACCTGTTTCCAAGAAGAGGGGAAAGAAGACGTTATGAAAGCTCAAAAAGTGACCTAGATATCGAAACAGACTACGAAGACCAAGCAGGTAATCTAAGAACGCGGCCGCCAG	Forward primer to amplify mNeonGreen with 100 bp homology to *CSE4*
AMo8738	GAATGAGTTCGCACTGGTGCAGGTACTTCAGTTTCCATTTCAGCTTCTTCTTCATTTTCTGTCTCGATTTCTCGCTTATTTAGAAGTGGCGCGCCTTCCTTGTATAATTCGTCCATACCC	Reverse primer to amplify mNeonGreen with 100 bp homology to *CSE4*

Table S6. PCR primers used in this study. Related to STAR methods.

Kinetochore Type	meiotic or mitotic	Binding Fraction	Preload Force (pN) ± SEM
Wild type	meiotic	0.52 ± 0.04<br>(*n* = 4)	4.38 ± 0.14<br>(*m* = 102)
	mitotic	0.53 ± 0.06<br>(*n* = 3)	3.10 ± 0.19<br>(*m* = 42)
*mcm21*Δ	meiotic	0.07 ± 0.00<br>(*n* = 2)	*no force measurement*
	mitotic	0.50 ± 0.00<br>(*n* = 2)	3.30 ± 0.16<br>(*m* = 57)
*iml3*Δ	meiotic	0.04 ± 0.01<br>(*n* = 2)	*no force measurement*
	mitotic	0.54 ± 0.05<br>(*n* = 2)	3.65 ± 0.31<br>(*m* = 23)

Table S7. Summary of rupture force experiments. Related to Figure 6.
Binding fractions indicate the fraction of beads that bound when held near the tip of a growing microtubule, expressed as mean ± σ from n experiments. The number of individual beads tested during each experiment ranged from 2 to 35.

Supplemental References

S1. Akiyoshi, B., Nelson, C.R., Ranish, J.A., and Biggins, S. (2009). Quantitative proteomic analysis of purified yeast kinetochores identifies a PP1 regulatory subunit. Genes Dev. *23*, 2887–2899.

S2. Shaw, W.M., Yamauchi, H., Mead, J., Gowers, G.O.F., Bell, D.J., Öling, D., Larsson, N., Wigglesworth, M., Ladds, G., and Ellis, T. (2019). Engineering a Model Cell for Rational Tuning of GPCR Signaling. Cell *177*, 782-796.e27.

S3. Chen, J., Liao, A., Powers, E.N., Liao, H., Kohlstaedt, L.A., Evans, R., Holly, R.M., Kim, J.K., Jovanovic, M., and Ünal, E. (2020). Aurora B-dependent Ndc80 degradation regulates kinetochore composition in meiosis. Genes Dev. *34*, 209–225.
